# Evaluation of Drug Permeation Enhancement by Using In Vitro and Ex Vivo Models

**DOI:** 10.3390/ph18020195

**Published:** 2025-01-31

**Authors:** Johan D. Steyn, Anja Haasbroek-Pheiffer, Wihan Pheiffer, Morné Weyers, Suzanne E. van Niekerk, Josias H. Hamman, Daniélle van Staden

**Affiliations:** 1 Centre of Excellence for Pharmaceutical Sciences, North-West University, Potchefstroom 2531, South Africa; dewald.steyn@nwu.ac.za (J.D.S.); anja.haasbroekpheiffer@nwu.ac.za (A.H.-P.); weyers.morne@gmail.com (M.W.); suzaevn@gmail.com (S.E.v.N.); sias.hamman@nwu.ac.za (J.H.H.); 2 Preclinical Drug Development Platform, Faculty of Health Sciences, North-West University, Potchefstroom 2531, South Africa; wihan.pheiffer@nwu.ac.za

**Keywords:** cell culture, drug delivery, ex vivo model, in vitro model, membrane drug permeation, permeation enhancers

## Abstract

Drugs administered by means of extravascular routes of drug administration must be absorbed into the systemic circulation, which involves the movement of the drug molecules across biological barriers such as epithelial cells that cover mucosal surfaces or the stratum corneum that covers the skin. Some drugs exhibit poor permeation across biological membranes or may experience excessive degradation during first-pass metabolism, which tends to limit their bioavailability. Various strategies have been used to improve drug bioavailability. Absorption enhancement strategies include the co-administration of chemical permeation enhancers, enzymes, and/or efflux transporter inhibitors, chemical changes, and specialized dosage form designs. Models with physiological relevance are needed to evaluate the efficacy of drug absorption enhancement techniques. Various in vitro cell culture models and ex vivo tissue models have been explored to evaluate and quantify the effectiveness of drug permeation enhancement strategies. This review deliberates on the use of in vitro and ex vivo models for the evaluation of drug permeation enhancement strategies for selected extravascular drug administration routes including the nasal, oromucosal, pulmonary, oral, rectal, and transdermal routes of drug administration.

## 1. Introduction

The achievement of therapeutic blood levels is a pre-requisite for the therapeutic efficacy of any drug entity. To prevent failure of approval of new drug entities as a result of low bioavailability during the clinical stages of the drug development process, the evaluation of pharmacokinetic properties early on during the preclinical stages is essential. High-throughput membrane permeation evaluation can be carried out using in vitro and ex vivo models. If poor membrane permeation is identified as a shortcoming of the new drug entity during these evaluations, then permeation enhancement strategies can be employed to improve its bioavailability [1].

**Figure 1:**
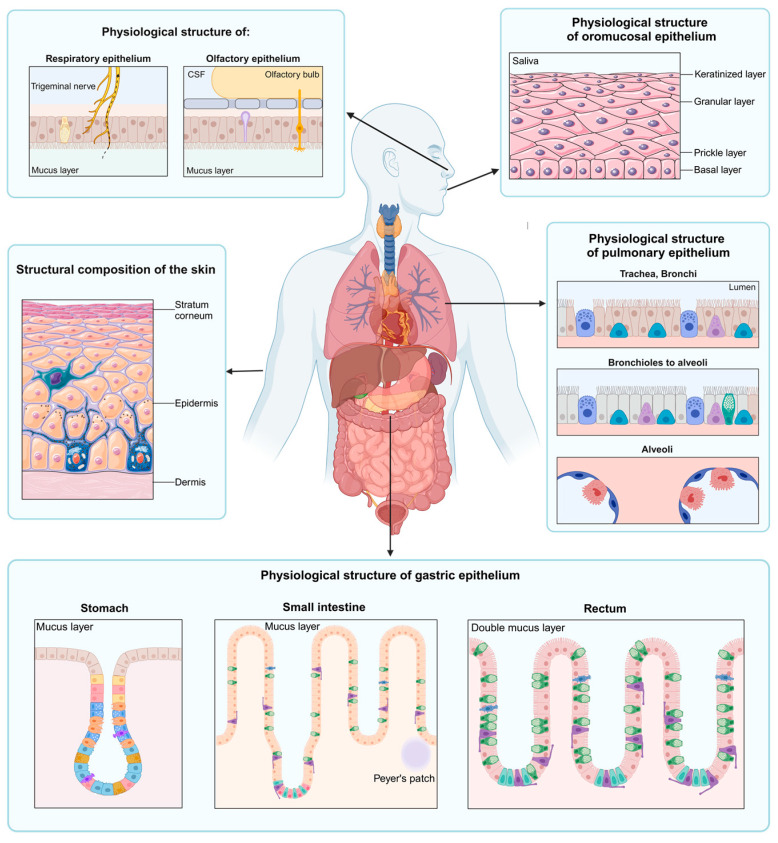
Illustration of skin composition and physiological structure of the epithelium of selected extravascular routes of drug administration [5,8,9,10,11]. Created in BioRender.

The discovery and development process of new drug entities consists of five stages. Stage 1 is the pre-discovery stage, where the disease mechanisms and drug target sites are explored and identified. Stage 2 focuses on drug synthesis, followed by a preclinical development stage (stage 3) that focuses on the elucidation of drug action/mechanisms and the evaluation of efficacy and potential toxicity using various in vitro/ex vivo and in vivo models. Positive drug activity and favorable toxicity results during stage 3 will also prompt progression to drug formulation or delivery device development. Stage 4 is then focused on drug activity and safety-related research in humans, followed by post-marketing surveillance and monitoring during stage 5 of the process [2]. However, many of these new drug entities do not progress further to the clinical research stages due to certain unique challenges that are associated with the successful delivery of these compounds to their respective target sites [2,3]. It is imperative to first select the most appropriate route of drug administration and then assess permeation across the appropriate membrane using representative in vitro/ex vivo models to estimate the rate and extent of drug delivery. For poorly permeable drugs, selected strategies are often used to improve/optimize the delivery of drug molecules, which include the implementation of selected formulation strategies, the use of permeation enhancers or bio-enhancers, and/or chemical modifications [4]. The use of suitable representative in vitro/ex vivo models to assess the extent of potential permeation enhancement during stage 3 studies provides a relatively low-cost, high-throughput alternative as compared to the use of in vivo models [4,5,6]. Additionally, from a research ethics-centered view, the use of in vitro/ex vivo models as screening tools before commencing with in vivo studies strongly complies with the 3Rs principle (reduction, refinement, and replacement) [7]. The most common extravascular routes of drug administration include the intranasal, oral, oromucosal, pulmonary, rectal, and transdermal routes. The physiological structure and composition of the biological membrane barriers of the extravascular routes of drug administration are illustrated in Figure 1.

**Figure 2:**
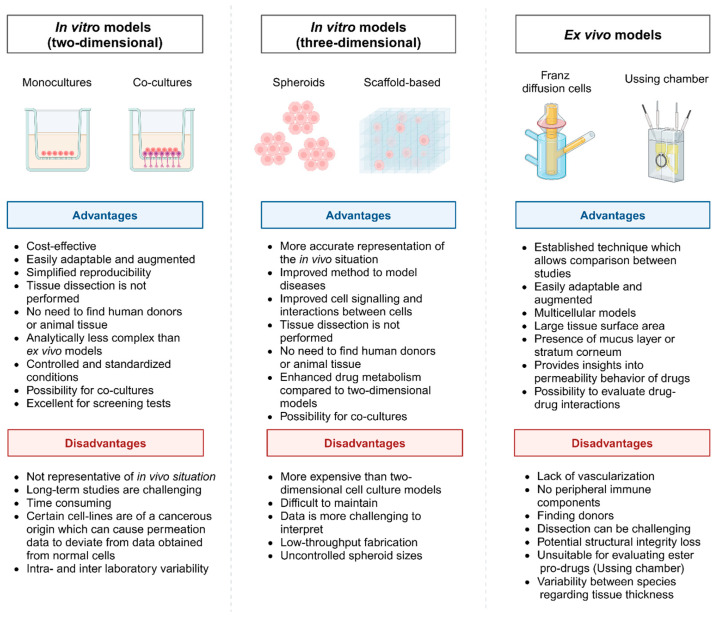
Diagram comparing the advantages and disadvantages of in vitro and ex vivo models [12,20]. Created in BioRender.

As seen in Figure 1, the physiological structure of the epithelium is unique for each extravascular route of drug administration. Drug delivery and potential permeation enhancement strategies can be evaluated via these routes of administration using a selection of representative in vitro/ex vivo models prior to commencing with in vivo studies, and these in vitro/ex vivo models are discussed in more detail in this review paper. Cell culture-based in vitro models evaluating permeation enhancement are well established, which generally allows the selection of suitable models to meet the specific aims of research projects [12]. Cell culture models can be divided into two-dimensional and three-dimensional models [12,13]. Two-dimensional models are most frequently used to study cellular responses to physical injury and drug exposure despite the literature reporting the superiority of three-dimensional cell culture models in providing a more reliable presentation of the in vivo situation [13,14]. The single-cell suspension (and, to a certain extent, the monolayer) nature of two-dimensional cell culture models lacks an accurate resemblance of interactions between cells and the extracellular matrix [12]. Two-dimensional cell culture models also ignore the impact of multicellular organization and do not sufficiently mimic the cellular microenvironment [13]. Meanwhile, three-dimensional cell culture models consider the transportation of growth factors, nutrients, and gas exchange [15]. This implies that three-dimensional cell culture models can present phenomena such as cellular proliferation, apoptosis, and differentiation, comparable to in vivo experiments [13]. Spheroids are self-assembled cell aggregates produced during three-dimensional cell culture experiments via static techniques like hanging drop and liquid overlay [16]. A downfall of spheroid models is the labor intensiveness, accompanied by the difficulty in controlling spheroid size and low-throughput production efforts [13]. However, assembled spheroids can also be cultured via dynamic techniques in bioreactors or microfluidic devices where fluid shear dynamics are controlled while mimicking a microenvironment resembling the in vivo environment to facilitate cell perfusion [17]. Microfluidic three-dimensional cell culture models gave rise to developments such as organ-on-a-chip systems and organoids to aid in disease-mimicking models to simplify drug development studies [18,19]. However, limited studies have reported utilizing microfluidic systems in drug permeation enhancement studies despite their frequent employment in common drug membrane permeation and uptake studies. Therefore, this review will provide examples of permeation enhancement studies performed with microfluidic systems where applicable, but does not give a comprehensive discussion on microfluidic systems for normal membrane permeation studies. Figure 2 considers the advantages and disadvantages of ex vivo and two-dimensional and three-dimensional in vitro models during permeation enhancement studies.

As seen in Figure 2, each permeation enhancement evaluation model presents unique advantages, but also challenges and disadvantages. Therefore, the model to be used in a specific study can be based on factors like the aim of the study, the allocated budget, the equipment and facilities available, the time frame, and the intended outcome. This review considers both established (commonly used models) and advanced models with an emphasis on the investigation of drug permeation enhancement strategies. Representative in vitro models for the assessment of intranasal and nose-to-brain delivery include RPMI 2650 cells and hCMEC/D3 brain endothelial cells (simulated BBB) [21]. Ex vivo models such as excised epithelial tissues obtained from various animal origins such as bovine, caprine, porcine, and ovine have been used to assess intranasal drug permeation in the presence of permeation enhancers [22,23,24].

The oromucosal route lacks the availability of standardized in vitro permeation models [25]. However, monolayer, two-dimensional, and three-dimensional cell culture models have been used as representative in vitro models of the oral mucosae for the assessment of drug permeation and permeation enhancement [26]. Buccal and oral mucosal tissues from various animal species such as rats, hamsters, rabbits, chickens, dogs, sheep, cows, monkeys, and pigs have been used as ex vivo models for the assessment of drug permeation for this route of drug administration [5,25,27].

Representative in vitro models that have been used to study pulmonary drug permeation include lung organoids and lung-on-a-chip models [28]. A limited number of in vitro cell culture models have been used in drug permeation enhancement studies [28,29], and their application is generally limited to studies other than permeation assessment such as cellular uptake, metabolism, toxicity, and medical device studies [28,30,31,32,33,34,35]. Calu-3 cells are grown on an air–liquid interface and are currently considered the in vitro model of choice for permeation studies pertaining to pulmonary drug delivery. Improvements in drug permeation using bio-enhancers (chitosan or selected fatty acids) or specialized formulations such as liposomes [36], nanoparticles [37,38,39], or inclusion complexes [40] have been studied using pulmonary in vitro models. Ex vivo pulmonary models are limited to isolated perfused lung (IPL) models and precise cut lung slices [28,41,42,43]. Rodent or rabbit lungs are generally used as ex vivo IPL models to study pulmonary drug delivery. IPL models have been used to study drug permeation using bio-enhancers [44], nanoparticle delivery systems [45], and co-administration with metabolic inhibitors [46].

In vitro models for the assessment of the oral route of drug administration for absorption from the gastrointestinal tract include Caco-2 cell cultures [47], co-cultures grown in monolayers in Transwell^®^ systems [48], and triple-culture models using three human cell lines (Caco-2, HT29-MTX, and Raji B cells) [49,50]. Additionally, three-dimensional intestinal transport models, based on human intestinal cells fused into different scaffolds, have more recently gained widespread use [51].

**Figure 3:**
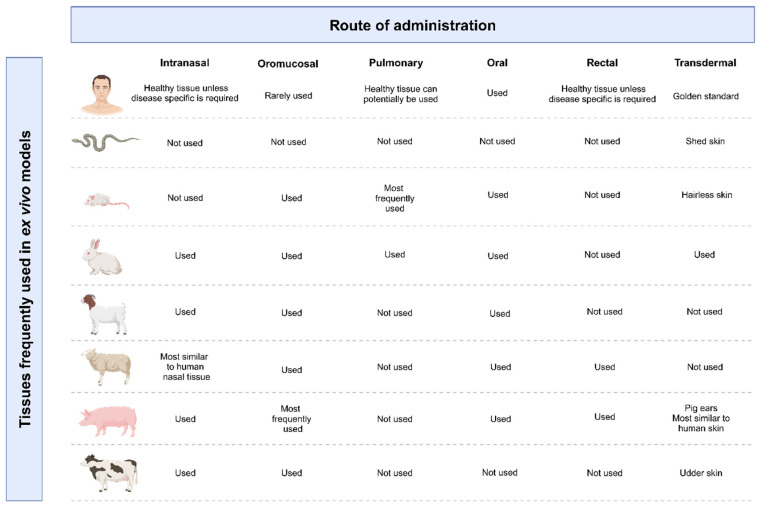
Tissue sources from animal species for ex vivo drug permeation enhancement experiments [10,20,27,44,55,56,57,58,59,60,61,62,63,64,65,66,67,68]. Created in BioRender.

Ex vivo models for the evaluation of oral drug permeation include the use of excised gastrointestinal tissues from various animal sources such as rats, sheep, and pigs [20,52]. Ex vivo pharmacokinetic studies involve the use of techniques such as everted intestinal sacs or excised tissue strips mounted in Ussing-type diffusion chambers [53]. Ex vivo models differ to some extent from in vitro cell culture-based models and are distinguished by various unique features such as the presence of a mucous layer, paracellular transport pathways, and transporter proteins, as well as drug-metabolizing enzymes [54]. However, interspecies variability due to physiological differences such as epithelial thickness can potentially cause data deviation with a lower correlation to that of human tissue. Hence, it is important to consider interspecies variability when selecting tissues to perform ex vivo permeation enhancement evaluations. Figure 3 provides an overview of tissues frequently used during ex vivo experiments for the extravascular administration routes considered in this review paper.

As demonstrated by Figure 3, the rectal drug administration route has the least ex vivo tissue harvesting possibilities for use in ex vivo models. Despite the relatively limited number of donor species, this route is still commonly evaluated using in vitro and ex vivo models due to the lack of reliable in vivo models to evaluate rectal drug absorption [69,70]. In terms of ex vivo rectal permeation studies, this study utilized human colon tissue removed during surgical procedures that did not involve gastrointestinal inflammatory conditions [65].

Cell culture-based models are also employed to evaluate the permeation of drugs across the skin. In vitro models for the assessment of transdermal permeation include two-dimensional monocultures such as the HaCaT cell model [71,72], scaffold-free three-dimensional models such as cell spheroids, and scaffold-based three-dimensional-engineered skin models which are produced using either natural or synthetic polymers or a combination of both types of polymers [73]. Furthermore, reconstructed human skin models have been used successfully for barrier function assessment and drug permeability evaluation [74,75]. Dermal ex vivo models include the use of dermatomed human skin and are considered to be the gold standard for the assessment of skin permeation [56,76,77,78]. Pig skin is also used frequently as a substitute for human skin, while the skin of other animal species such as primates, guinea pigs, rabbits, bovines, and reptiles has also been used in dermatological research, albeit with limited success [56,66].

This review provides an overview of in vitro/ex vivo models that have been used to study drug permeation enhancement for selected routes of drug administration. In some cases, depending on the available literature information, relevant drug examples and permeation enhancement approaches/strategies are also provided and discussed. This review not only comprehensively discusses conventional in vitro cell models, but also provides insights on and applications to the latest developments in the field. Furthermore, the diverse use of ex vivo tissue models in a variety of applications is outlined. The information provided in the text is also summarized and presented in Table 1 and Table 2 for quick reference purposes.

## 2. Nasal Route of Drug Administration

A route of drug administration that is receiving increasing attention for the delivery of drugs, including large-molecular-weight and peptide drugs, as well as vaccines, is the nasal (or intranasal) route of administration [4,79,80,81,82]. The nasal cavity is divided into various regions from which drug molecules can be delivered into the systemic circulation or directly into the brain (Figure 1). These regions include the respiratory region and olfactory region, which provide the opportunity for the delivery of drugs to produce either a local or systemic action [79]. This route also provides an opportunity to deliver drugs directly to the brain via the trigeminal nerve and olfactory neuron pathway, thereby bypassing the blood–brain barrier (BBB) and the blood–cerebrospinal fluid barrier (BCB), as well as avoiding first-pass metabolism [83].

The intranasal route of drug administration offers several advantages over oral drug administration, such as a relatively fast onset of action (i.e., within minutes). This route of administration also offers improved patient compliance since it offers a non-invasive drug administration alternative compared to the parenteral route. The highly vascularized nature, large surface area, and favorable permeability characteristics of the respiratory area are highly conducive to drug absorption, as this route of administration also avoids gastrointestinal and hepatic metabolism and enzymatic degradation in serum [4,79,84].

Several physical and biochemical obstacles exist that may limit the rate and quantity of drug absorption from the nasal cavity such as the mucus layer, mucociliary clearance, enzymatic degradation, epithelial layer composition, limited dosage volume, and the presence of tight junctions which exhibit relatively high transepithelial electrical resistance values [4,80,85]. Additionally, an important physicochemical factor controlling the diffusion of drug molecules across biological membranes is the lipophilicity of drugs. Drugs with a lipophilic nature (log P > 3) will generally diffuse across biological membranes at a greater rate and extent than those with a hydrophilic nature but are generally also more susceptible to pronounced enzymatic degradation which may ultimately cause a reduction in bioavailability [86]. To overcome some of these challenges and thereby improve the permeation of drug molecules after intranasal drug administration, various absorption enhancement strategies have been suggested, including mucoadhesive formulations, nanotechnology approaches, and co-administration with absorption/permeation enhancers (referred to as bio-enhancers when they are of natural origin) [4,81,87,88,89,90]. In order to investigate the efficacy of various absorption enhancement strategies, in vitro/ex vivo assessment in suitable preclinical models is necessary before commencing with in vivo studies. These preclinical models include in vitro cell culture and tissue-based ex vivo experimental models [89].

### 2.1. In Vitro Models for the Assessment of Nasal Drug Permeation Enhancement

The most widely used in vitro cell model to investigate permeation enhancement strategies across the nasal mucosa is the RPMI 2650 cell line (Table 1) [91], which was originally derived from human anaplastic squamous cell carcinoma in the nasal septum [3]. RPMI2650 cells are commonly grown in monolayers on membrane inserts in Transwell^®^ systems for drug permeation studies. However, cell monolayers do not completely represent the complex physiological and biochemical obstacles of the intact organs and epithelial barriers [92]. Two major drawbacks to RPMI cells are the difficulty of forming a confluent cell monolayer under submerged culturing conditions, specifically not expressing tight junctions, and the lack of goblet cell and ciliated cell expression [93]. These drawbacks can be overcome by culturing RPMI 2650 cells on an air–liquid interface, which produces a confluent, polarized cell monolayer with sufficient transepithelial electrical resistance values (indicating expression of intact tight junctions) [93,94]. The barrier function and physiological relevance of RPMI 2650 cell cultures can further be improved upon by co-culturing with various cell types [21,93,95]. RPMI 2650 cells have been co-cultured with human vascular endothelial cells to provide a better representation of the physiological barrier, representing both the nasal epithelium and the submucosal vascular endothelium [21,95]. Human nasal fibroblasts have also been used in the reconstruction of the nasal mucosa by using isolated human nasal fibroblast cells in a collagen matrix covered by RPMI 2650 cells, showing differentiated non-respiratory-like epithelium with permeation results comparable to excised nasal mucosa [93].

Despite the drawbacks, this cell line grown on an air–liquid interface has been used to investigate drug absorption mechanisms and toxicity [89] and has shown realistic permeability results for lipophilic, hydrophilic, and large-molecular-weight drugs [3]. RPMI 2650 cells were used to investigate the possible absorption enhancement of drugs by means of nanoparticle formulations [21,95], nanoencapsulation [96], and co-administration with bio-enhancers [22].

Akel and co-workers formulated insulin-loaded solid lipid nanoparticles and poly(lactic-co-glycolic acid) (PLGA) nanoparticles, uncoated and coated with chitosan, for nose-to-brain delivery [21]. These formulations were evaluated across an RPMI 2650 and human vascular endothelial cell co-culture to mimic the nasal barrier, and hCMEC/D3 brain endothelial cells to mimic the BBB. Improved mucoadhesion and sustained release were evident when using chitosan-coated formulations. The permeation of insulin across the nasal barrier co-culture was the highest from the chitosan-coated SLNs, followed by the chitosan-coated PLGA nanoparticles and the uncoated solid lipid nanoparticles and PLGA nanoparticles, with the lowest permeation observed for native insulin. Meanwhile, the permeation of native insulin across the brain endothelial model was higher than that of the chitosan-coated formulations, followed by the uncoated nanoparticles. The results suggest that the optimal nose-to-brain formulation contains both native insulin and insulin-loaded solid lipid nanoparticles coated with chitosan [21]. Another study evaluated the permeation of meloxicam across RPMI 2650 cell monolayers from meloxicam–human serum albumin nanoparticles containing various concentrations of poloxamer 407. A four-fold increase in meloxicam permeation from the meloxicam–human serum albumin nanoparticles, containing 14% poloxamer 407, was seen when compared to a meloxicam solution [95].

Nanoparticle-in-microparticle formulations containing transforming growth factor beta (TGF-β) were developed, comparing PLGA and lecithin to create nanoparticles. Drug transport from these formulations was investigated across air–liquid interface RPMI 2650 cell monolayers as well as cell monolayers grown from donated human nasal cells (representing different patient populations such as healthy individuals or individuals with asthma, polyps, or chronic rhinosinusitis with or without nasal polyps). The transport efficiency of TGF-β-lecithin was higher than that of the PLGA-containing formulation across RPMI 2650 cell monolayers. No significant differences in transport were observed for the different formulations when using cells from different patients [96].

Gerber and co-workers investigated the permeation-enhancing effects of different aloe species across RPMI 2650 cell monolayers and excised sheep nasal mucosa [22]. The apparent permeability of fluorescein isothiocyanate (FITC)-Dextran 4000 Da was significantly increased by all three aloe species at the highest concentration investigated. Epithelial layer damage was seen after exposure to *Aloe vera* materials and only slight damage was seen after exposure to *Aloe muth-muth*, for both the RPMI 2650 cell monolayers and excised sheep nasal mucosa [22]. Nerli and colleagues developed chitosan-coated niosomes, namely chitosomes, containing clonazepam. The transport of clonazepam was investigated across Caco-2 cell monolayers, showing a 10-fold higher apparent permeability (P_app_) of clonazepam from the chitosomes when compared to a clonazepam solution [97].

### 2.2. Ex Vivo Models for the Assessment of Nasal Drug Permeation Enhancement

Ex vivo models based on excised nasal mucosa from various animals have been widely used for membrane permeation studies because the excised tissue more closely mimics the composition of human nasal epithelial tissue than a monoculture cell layer [98]. The excised nasal epithelial tissue contains ciliated and non-ciliated cells, goblet and basal cells, and serous glands [99]. Excised tissue from several animal species has been used in the investigation of absorption enhancement strategies, such as bovine, caprine, porcine, and ovine tissue (Table 2). The absorption enhancement strategies that have recently been investigated using ex vivo models are mucoadhesive formulations [23,24,100,101], nanotechnology [23,24,59,83,100,102,103], and co-administration with absorption enhancers [22,23,24].

As an example, Kashif and colleagues developed and characterized eletriptan hydrobromide-laden pullulan nanoparticles, as well as a new biopolymer-based system of an eletriptan hydrobromide nanoparticle-laden mucoadhesive and thermoresponsive hydrogel [101]. The mucoadhesive and thermoresponsive hydrogel was formulated with optimized concentrations of chitosan, guar gum, and different poloxamers. The permeability of eletriptan hydrobromide from these formulations was investigated using frozen excised sheep nasal mucosa. The eletriptan hydrobromide nanoparticles showed an increased quantity of eletriptan hydrobromide penetration and an increased flux rate when compared to other nanoparticles incorporated in thermoresponsive hydrogels [101].

A study by Weyers et al. formulated and characterized chitosan microparticles containing FITC-dextran 4000 Da and piperine (a bio-enhancer) [24]. These microparticles were further formulated into thermosensitive gels. Drug delivery performance was investigated across excised sheep nasal epithelium dissected from frontal nasal concha, showing a 1.2-fold increase in FITC-dextran 4000 Da permeation with microparticles incorporating piperine [24]. As seen in Table 2, the diffusion of temozolomide from temozolomide nano lipid chitosan hydrogel formulations was investigated across excised goat nasal mucosa. The optimized formulation was compared to a chitosan hydrogel not containing nano lipids. The nanosized formulation increased the flux 2.4-fold [23]. Moreover, rotigotine was formulated in a nanoemulsion and a mucoadhesive nanoemulsion (using chitosan as the mucoadhesive compound). The percentage of drug permeated across excised goat nasal mucosa was determined, showing higher permeability after administering the mucoadhesive nanoemulsion when compared to the nanoemulsion alone [100]. Interestingly, another study evaluated insoluble particles of zaleplon which were nanosized with Soluplus^®^, poloxamer-188, sodium lauryl sulphate, and mannitol, to prepare nanocrystals. The prepared nanocrystals showed a 2.7-fold increase in the permeation of zaleplon through excised bovine nasal mucosa [103]. The literature also reports the formulation of bromocriptine mesylate- and glutathione-loaded nanoemulsions that were prepared using the ultrasonication technique. The bromocriptine mesylate- and glutathione-loaded nanoemulsion formulations showed a 3.4-fold and a 1.5-fold increase in permeation across the goat nasal mucosa, respectively, when compared to bromocriptine mesylate- and glutathione-containing suspensions [59]. Apart from nanoemulsions, a study documented a lamotrigine nanoliposome formulation, and the permeation was investigated across excised goat nasal mucosa. The optimized nanoliposome formulation resulted in a significantly higher percentage of drug transport and flux when compared to that of a lamotrigine suspension [83]. However, nanoemulsions remain a very promising permeation enhancement vehicle, as demonstrated by a study formulating an aripiprazole nanoemulsion as well as a mucoadhesive nanoemulsion (with the addition of Carbopol 971 to the nanoemulsion formulation) and comparing the permeation with an aripiprazole solution across the sheep nasal mucosa. The aripiprazole mucoadhesive nanoemulsion showed the highest steady-state flux and permeability coefficient [102].

## 3. Oromucosal Route of Drug Administration

Due to its convenience, the oral route of drug administration is the preferred way of drug treatment for patients and clinicians alike. Oromucosal administration does not require swallowing of the dosage forms, which further increases patient compliance and makes administration possible for pediatrics, geriatrics, and unconscious or non-cooperative patients. Additionally, it provides the opportunity to remove the preparation from the mouth when drug administration must be stopped due to adverse effects [104,105]. The oral cavity consists of the buccal mucosa, sublingual mucosa, soft palate, gingiva, hard palate, and the dorsal surface of the tongue [106]. The mucosa in the oral cavity protects underlying tissue from mechanical and chemical damage and acts as a physical barrier against invasion of microbes and foreign substances [107,108].

The oral mucosa consists of stratified squamous epithelium with intracellular spaces filled by lipids. These lipids are extruded from membrane-coating granules and organized in lamellae. Non-keratinized regions such as the cheeks, floor of the mouth, and lips are more permeable due to the less defined intercellular lipid composition. Lipids in these regions are typically in a non-lamellar, liquid phase, with only the occasional stack of lipid lamellae [109]. The non-keratinized lining mucosa, which includes the buccal and sublingual regions, is thinner than the masticatory and specialized mucosa on the inside of the cheeks and is therefore more suitable for drug delivery [27]. The cell and physical morphology of the oromucosal epithelium as a barrier against drug delivery has been reviewed previously and will therefore not be described in detail here [107,108,109]. With buccal administration, the preparation is placed on the inner cheek or between the cheek and gum, while sublingual administration refers to the placement of the preparation under the tongue [105].

When considering emergency medicinal treatment (epilepsy attack, hypoglycemic coma, opioid analgesics, etc.), oromucosal administration is a convenient route that is generally accepted by patients, parents, and caretakers as it maintains personal dignity, has fewer ethical considerations, and can be administered without prior technical training [105]. On the other hand, delivery of drugs via the oromucosal route of administration is restricted by the presence of a physical barrier in the form of stratified epithelia, intrinsic enzyme activity (proteases, cytochrome system, etc.), saliva, mucus, the relatively small surface area, the relatively small fluid volume available for dissolution, the amount of the formulation that might be swallowed, and whether the drug product was retained in the oral cavity. Other factors that might influence absorption include eating, drinking, smoking, and the presence of lesions or wounds [108,110,111].

The permeation of drug molecules across the buccal mucosa mostly entails passive diffusion by means of paracellular or transcellular transport [27]. Additionally, the permeation of drugs across the buccal mucosa to reach the systemic circulation marks the rate-limiting step of oromucosal drug delivery [112]. Properties such as surface area, the duration of drug release, and the effect of saliva wash-out can be regulated by careful dosage form and formulation selection [110]. In adults, the salivary flow rate ranges from 0.33 to 1.42 mL/min, the pH of the floor of the mouth and buccal mucosa is roughly 6.5 and 6.3, respectively, and the thickness of the buccal and sublingual mucosa is roughly 500–800 μm and 100–200 μm, respectively [105].

Sublingual and buccal formulations include tablets (immediate-release, sustained-released, controlled-release, chewable), solutions, suspensions, sprays, chewing gum, lozenges, strips, films, wafers, mucoadhesive formulations, gels, or orally disintegrating tablets [107,111]. The addition of permeation enhancers such as surfactants, fatty acids, or mucoadhesive biopolymers can increase drug absorption by disrupting intercellular lipids, improving drug distribution, improving drug solubility, interacting with cellular proteins, overcoming the enzymatic barrier, increasing the thermodynamic activity of drugs, reducing the viscosity of mucus, or altering the microenvironmental pH [108,109,113,114]. Alternative formulation strategies such as nanoparticles [115], liposomes, emulsions, microcontainers [116], patch-like structures or microwells, stimuli-responsive drug delivery systems [117], smart polymers [118], three-dimensional-printed scaffolds [119], microelectromechanical systems [120], and hybrid nanosystems [121] have been developed in more recent years.

The lack of standardization of in vitro and ex vivo permeation models for oromucosal drug delivery can be ascribed to using different animal species, the limited availability of tissue, varying cell culture conditions, and differences in experimental techniques [25]. Guidelines have been proposed for the development of a “quality by design” buccal permeation model. Variables such as measurement, environment, study conditions, permeation set-up, and receptor medium should be considered during model development and validation [122].

Due to the potential lack of accessibility to tissue, variability within tissue samples, and the high cost experienced with cell-based models, artificial models such as dialysis membranes, phospholipid-based biomimetic barriers (PAMPA, Permeapad^®^), the Phospholipid Vesicle-based Permeation Assay (PVPA), and the Artificial Membrane Insert System (AMI-system) have been developed [5,111]. These biomimetic barrier models provide a high-throughput screening alternative for mucosal preparations but should be incorporated in combination with an ex vivo model or an additional in vitro model to improve reliability [5].

### 3.1. In Vitro Models for the Assessment of Oromucosal Drug Permeation Enhancement

Monolayer, two-dimensional, and three-dimensional culture models have been developed to represent oral mucosae in drug permeation experiments [26]. Three-dimensional cell culture monolayer models which are cultured at the air–liquid interface allow for relevant exposure of the apical tissue surface, which more closely resembles in vivo characteristics of the mucosal epithelium than submerged monolayer cultures [106]. The TR146 human buccal carcinoma cell line forms multilayered non-keratinized stratified squamous epithelium with similar differentiation, morphology, structure, and enzymatic activity to normal human buccal epithelium [25,27]. HO-1-u-1 cell culture models originated from human sublingual squamous cell carcinoma cells presented with good correlation when compared to the P_app_ of β-blockers across the porcine sublingual mucosa and can be employed for in vitro screening of sublingual drug permeation [6].

Two-dimensional cell culture models include hamster cheek pouch primary cell cultures and the TR146 cell line. Three-dimensional, tissue-engineered models were found to be more representative of in vivo conditions and could be employed to determine tissue toxicity, irritancy, and transepithelial drug delivery [123]. These commercially available culture models include SkinEthic™ Human Oral Epithelium (TR146 buccal carcinoma-derived cell line) and Human Gingival Epithelium, EpiOral™ (normal oral keratinocytes), and EpiGingival™ [106].

When normal oral keratinocytes are cultured on top of normal oral fibroblasts, the models are referred to as oral mucosa equivalents. As normal oral keratinocytes exhibit interindividual variability and a brief lifetime, TERT-2 immortalized oral keratinocytes have been developed to overcome this limitation [25]. Co-culture systems have been developed for both monospecies and multispecies 2D models and 3D tissue-engineered models that closely mimic the oral cavity while including biofilms and/or planktonic bacteria. These systems can be implemented to evaluate interactions between cells and evaluate disease progression when relevant [124]. A mucus-on-a-chip microfluidic device can be utilized to determine both mucoadhesion and permeation, elucidating mucus binding interactions [125].

Table 1 and Table 2 provide a summary of permeation enhancement strategies for enhanced oromucosal drug delivery, together with the relevant models employed to evaluate the specific permeation enhancement strategies. Examples of drug permeation enhancement studies include the work by Chen and co-workers, who developed a self-assembled liposome system to deliver carvedilol to the buccal mucosa. Carvedilol was successfully delivered across excised porcine buccal mucosa and a TR146 cell model. The formulation increased bio-adhesive performance and presented with better permeation compared to a carvedilol suspension [126]. Tissue-engineered oral mucosal equivalents of normal human oral fibroblasts and immortalized oral keratinocytes FNB6-TERT, as well as excised porcine cheeks, were used to evaluate the sustained release of clobetasol-17-propionate from electrospun nanofiber bilayer mucoadhesive oromucosal patches. Prolonged drug release was obtained in both models [123].

### 3.2. Ex Vivo Models for the Assessment of Oromucosal Drug Permeation Enhancement

Most drug permeability studies for preclinical oromucosal drug delivery prediction are performed ex vivo, which can be explained by the accessibility of tissue, low cost, maintenance of experimental conditions (such as temperature and pH), easy sample analysis, and lower time consumption compared to in vivo studies. Buccal absorption and perfusion tests (swirl and spit) are variable due to drawbacks such as accidental swallowing, and in vivo studies are too expensive and laborious [27].

Human buccal mucosa is scarce; therefore, several animal species have been used as alternative sources for buccal mucosa, such as rats, hamsters, rabbits, chickens, dogs, sheep, cows, monkeys, and pigs. Rats and hamsters have keratinized epithelium, while rabbits have non-keratinized epithelium, but a relatively small tissue area is available due to the small size of the oral cavity. Monkey, ape, and dog oral cavity mucosae are non-keratinized, but thinner than the human oral cavity mucosa. The oral cavity mucosa of pigs is very similar to that of humans and is non-keratinized and similar in structure, thickness, cellular morphology, and lipid composition [5,25,27]. A comparative study of metoprolol permeation across excised human and porcine buccal mucosa indicated lower permeability across human buccal tissue than porcine, but presented a clear correlation of P_app_ values between these two species [127]. The intrinsic permeability coefficients of the porcine buccal mucosa have been summarized, and parameters with an effect on permeability such as permeant lipophilicity and molecular size have been investigated to create a database of permeability coefficients [128].

The esophageal mucosa has been used as an alternative for the buccal mucosa as it provides a larger surface area, is easy to excise, and comprises the same epithelium and intercellular lipid composition as the buccal mucosa [5,129]. Acyclovir gel with controlled release across dialysis membranes indicated a positive linear correlation to permeation across the chicken pouch membrane [130]. Other alternative membranes include the chick chorioallantoic membrane or human vaginal mucosa. However, the chick chorioallantoic membrane has a very small surface area, and the human vaginal mucosa is extensively influenced by age [5]. The rat buccal mucosa was used as a model membrane to evaluate the potential co-delivery of rizatriptan benzoate and propranolol hydrochloride from a mucoadhesive buccal film [68]. The rabbit buccal mucosa was implemented to evaluate an oral mucoadhesive film containing palonosetron. A biphasic drug release profile and increased flux were observed [62]. The delivery of a febuxostat self-nano-emulsifying system-loaded sublingual fast-dissolving film was evaluated across excised sheep sublingual tissue. A faster onset of action and increased permeation were achieved [131].

The in vitro, ex vivo, and in vivo buccal absorptions of metoprolol as a function of buffered pH (at 7.4, 8.5, 9.0, and 9.5) were compared. The results of the permeability studies showed a correlation (r^2^ = 0.92) between the in vitro TR146 cell culture and ex vivo porcine buccal mucosa in a modified Ussing chamber. In addition, a level C in vitro–in vivo correlation (r^2^ = 0.98) was observed between in vitro predictions and the in vivo bioavailability of metoprolol administered buccally to mini-pigs, indicating the prediction potential of these in vitro models [132].

## 4. Pulmonary Drug Delivery

The pulmonary route of drug administration refers to the administration of drugs to the lung to mainly treat respiratory diseases locally in the lung (e.g., asthma, lung cancer, and chronic obstructive pulmonary disease), but can also be used to deliver drugs systemically [133]. Since drugs are directly administered into the site of action for local action (i.e., respiratory tract and lung), the amount of drug needed for a therapeutic effect is usually less than that needed for oral administration [134]. The advantages of inhalation therapy include direct delivery into the target organ for local action. However, systemic absorption may also occur due to the large and highly vascularized surface area with a relatively thin air-to-blood barrier [135,136]. Additionally, enzymatic degradation of drugs also occurs to a lesser extent in the lungs compared to the oral route of administration [137,138].

There are biological barriers against drug absorption in the lungs that form part of the first defensive line against the accumulation of pathogens, allergens, and particulate matter in the lungs [139], which can also delay and reduce the absorption of drug molecules. These include the mucociliary escalator, alveolar macrophages, and respiratory mucus, which are secreted by the respiratory tract epithelium [139,140]. Additionally, at the apical junction, complexes between epithelial cells regulate and restrict paracellular permeability [138,141]. Therefore, the rate-limiting step of pulmonary drug delivery is the capacity of molecules to reach the deep lung tissue to enforce therapeutic efficacy [142]. The advances in pulmonary drug delivery, including devices, applications, and strategies to improve formulations, have previously been reviewed [35,36,90,139,143,144,145]. These approaches to enhancing pulmonary drug delivery were confirmed using various biological models, which include in vitro and ex vivo approaches.

Due to the complexity of the lung with its specialized structures and cell types for specific physiological functions, it is problematic to elucidate the complete drug absorption and disposition processes after pulmonary administration [42,146]. To investigate and comprehend the processes involved in drug absorption and disposition of inhaled drugs, various in vitro and ex vivo models have been used [42,43,146]. These models serve distinct purposes and can be used to investigate specific processes and mechanisms within the lung, and they often express one or more of the anatomical or physiological properties that are considered important for the prediction of pulmonary drug delivery [42,146]. Thus, it is important to be aware of each model’s intended application and its limitations. Pulmonary models have advanced in recent years, but future research should focus on further refining and validating both in vitro and ex vivo models to mimic the complex composition of the lung and respiratory system more accurately [35,41,144,146].

### 4.1. In Vitro Models for the Assessment of Pulmonary Permeation Enhancement

Numerous in vitro models that have been used for pulmonary drug delivery studies include traditional cell cultures and lung-on-a-chip models [28]. Some in vitro cell models have been used in drug permeation enhancement studies [29,147], but are mainly used in cellular uptake, drug efficacy, metabolism, pharmacodynamics, toxicity, and medical device studies [28,30,31,32,33,34,35]. The “golden standard” of permeation studies involving pulmonary drug delivery is the use of Calu-3 cells grown on an air–liquid interface (Table 1), but there are no current regulatory guidelines in place for standardized testing [89]. Other cell lines grown on an air–liquid interface are also used, such as 16HBE14o and VA10 cells (Table 1). These models allow for air-to-liquid transport studies but lack the complexity of the target organ—apart from the non-physiological conditions, they lack the complex 3D environment of tissue and the complex architecture of the lung [148,149]. Cells grown on an air–liquid interface do, however, differentiate into morphologically and histologically relevant airway epithelial cells, which provides more relevant results in inhalation studies that complement in vivo results better, compared with submerged cell cultures [148,149,150].

Air–liquid interface cell culture models can indicate enhancement of drug permeability by measuring the percentage of drug transported or P_app_ across the cell monolayers. Improvement of drug permeation can be achieved by incorporating bio-enhancers like chitosan or fatty acids, or by delivery system strategies, like liposomes [36], nanoparticles [37,38,39], or inclusion complexes [40] (Table 1). These permeation enhancers increase drug permeability by improving paracellular drug transport by means of tight junction modulation, or by increasing the contact surface area (Table 1). Apart from using in vitro cell culture models to evaluate improved drug delivery, synthetic cellulose membranes have also been used (Table 1) to gauge drug diffusion.

Lung-on-chip models mimic a wide range of physiological and mechanical parameters of in vivo respiration [151]. This is achieved by replicating airflow and pressure dynamics and breathing mechanics, incorporating a diversity of cells, and mimicking the vascularity of the lung [151]. Thus, lung-on-chip models are a much more complex system compared to the gold standard air–liquid interface cell monolayer models. The complexity allows for more advanced investigations into the behavior of inhalation studies and how the physiological and mechanical barriers may impact drug uptake [28]. Lung-on-chip models are mostly used for drug efficacy, toxicity, and disease-specific studies [152,153,154,155,156], and there are limited drug permeation studies in the literature [151,157,158]. The potential to use these models in drug development and screening has been highlighted in previous publications [159,160,161]. Moreover, these models have potential in applications in drug permeation enhancement studies.

### 4.2. Ex Vivo Models for the Assessment of Pulmonary Permeation Enhancement

Ex vivo respiratory models for inhalation drug research are limited to IPL models and precise cut lung slices [28,41,42,43]. IPL models make use of rodent or rabbit lungs and are the most widely used ex vivo models in pulmonary drug delivery studies. These ex vivo models are primarily used for drug delivery and transport studies, efficacy studies, pharmacokinetics and metabolism studies, and elucidating biochemical functions from pulmonary drug delivery [30,35,44,45,46,134,162,163,164,165]. Precise cut lung slices are used for drug efficacy and toxicity studies [166,167,168,169]. Rat lungs are the most used in IPL-type models (Table 2). The main limitations of ex vivo pulmonary models are species differences, which result in difficulty translating findings into clinical settings [28,170]. Ex vivo lung models are, however, more complex than in vitro models, retaining the 3D structures of the donor lung. They also allow the study of specific variables under controlled conditions in real time [171,172] while reducing the unnecessary use of animals. Formulation strategies such as particle coating with bio-enhancers [44] or nanoparticle delivery systems [45] to increase paracellular transport and to increase cellular uptake have been tested using IPL models (Table 2). Additionally, co-administration of metabolic inhibitors has been investigated [46], where the effect of active transporters was also reduced and a subsequent increase in lung absorption was achieved (Table 2). Isolated perfused rat lungs were also used to compare carriers intended for medical devices, such as inhalation suspensions versus dry powder formulations [164], where the changes in some physicochemical properties proved to increase drug absorption (Table 2). Complex in vivo models were more regularly used after in vitro investigations were finalized to investigate drug delivery, permeation, and pharmacokinetics [173,174,175,176,177].

## 5. Oral Route of Drug Administration

The literature frequently reports the oral route of drug administration as the most desirable route as attributed to its convenient administration, elevated patient adherence, decreased manufacturing costs, and favorable safety profiles [178,179,180]. Despite these advantages, oral drug administration can be challenging as drugs and dosage forms are exposed to divergent physiological environments when traveling through the gastrointestinal tract, which can impede drug absorption [181]. Importantly, drugs are exposed to an initial strongly acidic gastric environment with an approximate pH of 1.2 and conclude the enteral route with a pH of 7.0 in the colon [180]. Additionally, drug absorption can be hindered in the gastrointestinal tract by digestive enzymes, tight junctions, efflux transporters, and mucosal barriers protecting the underlying epithelium where drug absorption should take place [180,182]. In terms of oral drug administration, drug solubility in the gastrointestinal fluid and permeability across the intestinal mucosa can be defined as the rate-limiting steps despite the nature of the administered dosage form [183].

Oral drug administration aims to mediate drug absorption into the systemic circulation or lymphatic system to establish a therapeutic effect. Currently, oral drug administration research is focused on strategies such as targeted oral drug delivery, implying that dosage forms are designed to compensate for poor drug properties by protecting drugs until transport across mucosal surfaces or uptake into the lymphatic system can be achieved [179,182,184]. Examples include the development of pH-responsive drug carriers, lipid-based drug delivery systems targeting the lymphatic system, and innovative mucus-permeating formulation strategies [178,179,180,182,184,185]. Drugs intended for oral administration should demonstrate the capacity to withstand degradation within the divergent gastrointestinal environment, permeate enteric epithelium, bypass blood-mediated clearance, and ultimately reach the target site to perform a therapeutic action [180]. Therefore, in the early stages of the development of a new chemical entity or oral dosage form, in vitro permeability assays provide valuable and vital information about the drug in terms of its transport capacity across intestinal barriers [186]. Moreover, intestinal-based ex vivo tissue models are relatively inexpensive and offer an alternative to simplified in vitro models. These models more closely mimic physiological and anatomical properties and maintain biochemical functions due to the preservation of tissue viability. This characteristic contributes to the acquisition of detailed information on how drugs will perform in the in vivo environment. Ex vivo transport studies mainly use excised animal tissues to predict human intestinal absorption [187]. Additionally, epithelial tissues from different intestinal regions can be used to elucidate drug permeability at a targeted site [188].

### 5.1. In Vitro Models for the Assessment of Gastrointestinal Permeation Enhancement

The main advantages of in vitro cell culture models include their relative simplicity, resemblance to the human gastrointestinal physiological environment, and potential to predict interactions as would occur in vivo [189]. Cell-based in vitro models can be used for conducting mechanistic drug transport studies [189]. The Caco-2 cell line is the most widely used cell line to simulate the intestinal epithelial barrier. The descriptions, utilization, advantages, and limitations of Caco-2 cells in evaluating the intestinal transport of drugs have been thoroughly reviewed elsewhere [47]. A more advanced concept is the use of co-cultures grown in monolayers in Transwell^®^ systems. The Transwell^®^ intestinal system consists of a polyester/polycarbonate membrane (10 μm) with a range of pore sizes on which intestinal epithelial cell monolayers can be cultured, which separates the apical compartment from the basolateral compartment, which represent the intestinal lumen and submucosa, respectively [48].

Although co-culture models have been extensively used as in vitro tools for the evaluation of intestinal drug transport, neither mucus-secreting nor folate-associated epithelial cells nor folate-associated epithelium models simulate the intestinal epithelial layer entirely. Considering the importance of three main types of epithelial cells in intestinal physiology (e.g., absorptive enterocytes, mucus-secreting goblet cells, and antigen-delivering M cells) [49], a triple-culture in vitro cell model with three human cell lines (Caco-2, HT29-MTX, and Raji B cells) was developed to investigate the intestinal transport of insulin in solution and encapsulated within nanoparticles [50,190]. This triple-culture model was used successfully to evaluate the intestinal permeability of several drug molecules, including enhanced transport of insulin by formulating it into nanoparticles [191]. The findings of this study suggested that a sophisticated triple-culture model could be a suitable tool for elucidating the transport mechanisms of drugs [192].

Schimpel and co-workers also developed a triple-culture cell model (Caco-2/HT29-MTX/Raji B) to investigate the intestinal transport of polystyrene nanoparticles [193]. The resulting permeability data showed a good correlation between the in vitro triple-culture cell model and an ex vivo porcine intestinal mucosa model, suggesting that this triple-culture cell model is a reliable in vitro model for studying particle uptake [193]. Three-dimensional intestinal transport models that mimic the morphology and physiology of human gastrointestinal barriers have recently attracted the attention of researchers. Three-dimensional intestinal cell models are typically based on human intestinal cells fused into different scaffolds (promoting cell proliferation and differentiation) [51]. Compared with the classic Caco-2 cell monoculture model, this model more closely mimics the native intestinal layer with a higher correlation coefficient, representing a better predictive tool for the study of drug intestinal transport [194].

The gold standard model for drug permeability assays comprises a flat polarized monolayer of enterocyte-like Caco-2 cells. It only represents intestinal mucosal epithelium and one cell type, the enterocytes, which misses the characteristic three-dimensional topographical features of the native tissue. Compared with the human intestine, the Caco-2 model has different expression levels of drug transporters and forms a tighter barrier, which can lead to low accuracy when predicting drug intestinal permeability [195].

A recent study tested the impact of the improved features observed in a bio-printed model of the intestinal mucosa for drug intestinal permeability experiments [196]. The improved model hosted three-dimensional intestinal villi and was compared with a flattened villi model. The study employed three model drugs with different permeability characteristics, namely metoprolol, atenolol, and colchicine. Metoprolol showed similar apparent permeability values for both the villi and flat bio-printed models, which suggested that the transcellular pathway is not affected by the three-dimensional architecture of the model. The most significant differences in permeability were found with colchicine due to lower genetic expression of P-gp and multidrug resistance-associated protein 2 in the three-dimensional villus-like model. The lowered activity of P-gp-related efflux resulted in improved colchicine permeation in comparison to the flat model. Thus, these permeability results are consistent with the flat model [196]. Moreover, the study investigated permeability in a comparative flat vs. three-dimensional bio-printed villi mucosal model, where the permeability assay of rhodamine 123 was performed from the apical to the basolateral direction and vice versa. The three-dimensional bio-printed villi mucosal model demonstrated that the drug transport was almost six times higher in the apical-to-basolateral direction. This difference could most likely be attributed to the larger surface area of the villi model in comparison to the flat configuration and decreased P-gp activity in the flat model. When comparing the results in the basolateral-to-apical direction, it was clear that the P_app_ values that were obtained using the villi model were very similar in both directions, demonstrating an efflux ratio value close to 1. In the flat model, the transport of rhodamine 123 was significantly increased in the basolateral-to-apical direction, with an efflux ratio that was four times higher compared with the apical-to-basolateral direction. The results suggested a significant reduction in the activity of the P-gp transporter in the three-dimensional bio-printed villi mucosal model [196]. The results of the study showed that culturing cells in a more physiologically representative environment with a stromal compartment and three-dimensional architecture can lead to more predictable and repeatable permeability outcomes [196].

However, conventional in vitro cell-based models, including three-dimensional intestinal models, are employed as static models and therefore lack the dynamic and active microenvironment that exists in vivo. To ameliorate these limitations, in vitro microfluidics-based systems, including gut-on-a-chip and human–microbial crosstalk (HuMiX), have emerged as advanced cell culture models for studying drug transport across intestinal barriers. These models utilize microfluidic technology for in vitro cell culturing, which includes reproduction of the three-dimensional topology, dynamic environment, and gut microbiome that are observed in the human intestine [197].

The most common design for gut-on-a-chip microdevices consists of a porous membrane that supports a monolayer of intestinal epithelium cells that separates two compartments, simulating the intestinal lumen and blood circulation, respectively. Kim and co-workers developed a human gut-on-a-chip system that mimicked intestinal luminal fluid flow and peristalsis-like motions [198,199]. The model was later used to study peristaltic movements in a three-dimensional human gastric organoid system [198]. The study observed peristaltic movements of the model compound FITC-dextran through the human gastric organoid system. The study reported steady-state luminal flow over a sustained period of more than 30 min, thus demonstrating proof of concept for long-term delivery and observation of luminal agents.

### 5.2. Ex Vivo Models for the Assessment of Gastrointestinal Permeation Enhancement

Ex vivo studies are based on experiments performed on tissue excised from organisms in a controlled external environment that resembles natural physiological conditions [20]. One gastrointestinal ex vivo model is known as the gastrointestinal tissue robotic interface system (GI-TRIS), which is an interfacial device that has recently been developed to allow high-throughput evaluation of drug membrane permeation [200,201]. A recent study by von Erlach and co-workers investigated the permeation of oxytocin across freshly isolated jejunum and ileum tissue using a whole-tissue robotic interface system [201]. The study also included the transport assessment of a model compound, FITC-dextran, across the epithelium of other segments of the gastrointestinal tract than the jejunum, including the stomach, duodenum, and colon. The intestinal transport data obtained by the GI-TRIS system were compared to intestinal absorption data from humans based on previously published data for more than 50 drugs. The study reported an excellent correlation between the average intestinal transport values obtained by the GI-TRIS system and the reported human data. A Spearman correlation coefficient of 0.906 was demonstrated with near-perfect in vivo predictability [201]. A study by Gandia et al. (2004) aimed to investigate a new ex vivo/in vitro model to study drug permeability by using three absorption markers [202]. The researchers used perfused everted intestinal segments of rats, and the markers were as follows: antipyrine for passive transcellular diffusion, mannitol for paracellular diffusion, and digoxin as a P-gp substrate. The results demonstrated a mean P_app_ of 6.07 (±0.99) × 10^−5^, 8.79 (±0.28) × 10^−6^, and 3.1 (±0.85) × 10^−5^ cm/s for the markers, respectively. Thereafter, the study used the data from the everted gut sac method to determine which excipients effectively enhanced the permeation of P-gp substrate drugs across the intestinal mucosa. Selected excipients were further evaluated in vitro to determine possible in vitro/in vivo correlations. This demonstrated that several of the excipients evidently modified the pharmacokinetics in vitro but failed to increase the overall oral bioavailability (as measured by the area under the curve) [89,202]. Moreover, a recent study by Doppalapudi et al. (2023) studied the effects of bromelain on glucose uptake using the everted gut sac technique [203]. The study added glucose at different concentrations into the mucosal compartment liquids and simultaneously added bromelain to simulated gastric fluid. The pattern of glucose uptake ex vivo was analyzed using Michaelis–Menten and Lineweaver–Burk plots, and the results showed that bromelain increased the permeability of glucose in the ex vivo model. The study came to this conclusion with the comparison of K*m* and V*max* values between the study groups and control groups. The K*m* decreased while the V*max* remained unchanged in the presence of bromelain at a dose of 10 μg/mL, but at higher doses (up to 20 μg/mL), the V*max* increased while the K*m* remained constant when compared with the control groups [203].

The literature reports that co-polymers can also serve as possible bio-enhancing formulations with favorable biocompatibility. A study by Gao and co-workers studied the potential impact of 1, 2-Distearoly-sn-glycero-3-phosphoethanolamine-poly (ethylene glycol) (DSPE-PEG), a type of phospholipid–PEG copolymer, on intestinal absorption and related mechanisms of berberine permeation in rats [204]. The effects of DSPE-PEG on the extent of intestinal absorption were investigated in situ (closed loop), and the probable mechanisms were explored based on its impact on P-gp function and tight junction integrity using an in vitro diffusion chamber system. The co-polymer formulation, DSPE-PEG polymer (1.0%, *w*/*v*), demonstrated significant enhancement action on berberine absorption in rats without any obvious membrane toxicity. The mechanistic studies demonstrated that the DSPE-PEG polymer did not directly control intestinal P-gp function, but rather down-regulated the expression of tight junction-related proteins, which in turn resulted in a decrease in tight junction integrity between intestinal epithelium cells, which consequently resulted in increased paracellular absorption of berberine in rats [204].

## 6. Rectal Route of Drug Administration

As the final section of the gastrointestinal tract, the rectum is considered a multifaceted alternative route to oral drug delivery as systemic or local drug absorption can be achieved following rectal drug administration [70]. The rectum comprises columnar epithelium together with goblet cells that are responsible for mucus secretion. Rectal columnar epithelium is covered by a double mucus layer, with the inner layer working as a filter system to guard underlying rectal epithelial cells against bacteria. In humans, the second mucus layer, generated by protease activity to convert the stratified inner mucus layer to an unattached outer mucus layer, can be found approximately 200 µm away from the underlying epithelium [205]. In contrast to the small intestine, a smaller surface area for drug absorption is presented by the rectal area since villi and microvilli are absent from the luminal rectum surface [206]. Also, rectal fluid presents a smaller volume for drug dissolution, which can be described as a rate-limiting step of rectal drug absorption. The presence of fecal matter in the rectum can also impede dissolution, impact dosage form stability, and decrease the contact time between the drug(s) and rectal mucosa. Drugs can also be retained in mucus if permeation enhancement strategies fail to effectively cross mucosal barriers [206]. In terms of perfusion, blood is removed from the rectal area via the superior, middle, and inferior rectal veins [206]. The inferior and middle veins can provide access to the systemic circulation. Meanwhile, the superior rectal vein can mediate drug entry into the portal venous system [206]. Moreover, the rectal area is known for its decreased enzymatic activity, which makes it a suitable route of administration for drugs that exhibit instability upon oral administration, drugs that are excessively susceptible to first-pass liver metabolism, drug entities with poor oral absorption, and drugs notorious for causing gastric irritation [70,206]. Therapeutically, the rectal route is favored above oral administration if patients are vomiting, have difficulty swallowing, or are unconscious or if drugs have unpleasant organoleptic properties [70,206]. However, rectal drug administration is generally reserved for situations where oral and intravenous drug administrations are considered unattainable as patients tend to avoid rectal drug administration [206]. Therefore, the rectal route has not received as much scientific investigation as the oral route of administration.

### 6.1. In Vitro Models for the Assessment of Rectal Permeation Enhancement

The number of rectal drugs currently under research development and clinical investigation is hindered by the lack of a reliable in vivo experimental model [69,70]. Therefore, in vitro and ex vivo models are highly important during the development of rectal dosage forms to predict permeation enhancement. The literature reports the use of vaginal cell culture-based models to predict drug permeation enhancement for rectal tissue as similar dosage forms (i.e., suppositories, creams, and gels) are conventionally used to treat localized pathologies in both the rectum and vagina. Both are considered body cavities fulfilling different physiological functions, as the vaginal channel is imperative to reproduction and the rectum acts as an expulsion organ [207]. However, while utilizing vaginal cell-based in vitro models can provide insight into transmucosal drug permeation, the physiological structures of rectal and vaginal tissue differ in structure and thickness. Vaginal tissue harbors a thicker layer of stratified squamous epithelium. Meanwhile, the rectal mucosa comprises a single columnar epithelial cell layer reported as being up to 8-fold thinner than vaginal epithelia. Due to the reduced thickness of rectal epithelium, hydrophilic drugs superiorly permeate rectal epithelium compared to vaginal epithelium [208]. Ham and co-workers have reported utilizing a commercialized vaginal cell culture model to evaluate permeation from a microbicide gel intended for both rectal and vaginal application. During this study, the EpiVaginal™ Human Vaginal-Ectocervical three-dimensional tissue model demonstrated consistent microbicidal release and permeation into the cultured tissue from DuoGel™ formulations over a 4 h period [65]. However, the literature generally reports the use of Caco-2 cells to investigate rectal tissue permeation. For instance, a study by Wang and co-workers considered the permeability of 5-fluorouracil included in a complex with hydroxypropyl-cyclodextrin to enhance the efficacy of a 5-fluorouracil thermo-reversible gelling film [209]. Caco-2 cells were employed to evaluate rectal drug absorption. Transepithelial electrical resistance values were obtained to understand the mechanism rendering enhanced permeation by considering the integrity of cellular tight junctions. This study reported that enhanced permeation of 5-fluorouracil was achieved via enhanced drug solubility driving intracellular transport, as displayed by the in vitro Caco-2 cell model [209]. Moreover, a study by das Neves and co-workers investigated the use of nanoparticle formulations and conducted transport studies using a Caco-2 cell line [210]. Specifically, intracellular/cell-associated drug levels were assessed by determining the amount of dapivirine taken up into Caco-2 cells upon incubation with nanoparticle dapirivine and free dapivirine [210]. The findings concluded that nanoparticles can provide mildly enhanced absorption of dapirivine compared to its free form. Additionally, nanoparticles modified with cetyl trimethylammonium bromide provided increased permeation compared to other considered nanoparticles due to the intensified reaction between negatively charged cell membranes and positively charged cetyl trimethylammonium bromide-modified nanoparticles, which can improve dapirivine absorption [210].

### 6.2. Ex Vivo Models for the Assessment of Rectal Permeation Enhancement

The same study evaluated the ability of nanoparticles to associate and penetrate the female pig rectal mucosa upon 2 h incubation with rhodamine 123-loaded nanoparticles [210]. Fluorescence microscopy images revealed that all nanoparticle formulations were able to penetrate rectal mucosal tissues. Also, confocal microscopy demonstrated the colocalization of nanoparticles and membranes in the same plane, thus indicating that nanoparticles were indeed embedded in the mucosa and not simply deposited above or below the tissue as a result of dragging during tissue slicing [210]. Another study also employed the pig rectal mucosa to evaluate diltiazem hydrochloride absorption from rectal gels containing drug-loaded microsponges [63]. Permeability profiles revealed enhanced permeation from gels comprising methylcellulose in comparison with poloxamer 407-containing gels, as attributed to its mucoadhesive properties. Interestingly, changes in permeation profiles were also observed in the course of diltiazem hydrochloride permeation from conventional gels occurring between 1 and 2 h, compared with 3 and 6 h for gels containing microsponges. This can be attributed to the time needed to reach an equilibrium between the rectal mucosa and the gels before enabling diltiazem hydrochloride permeation [63]. According to the literature, similarities exist between the rectal mucosae of humans and pigs. This implies that porcine rectal tissue can be an appropriate substitute for human rectal tissue as human rectal tissue can only be harvested after colorectal surgery or from cadavers which are subjected to strict ethical consideration [211]. Apart from pig rectal tissue, a study reported utilizing the sheep intestinal mucosa to evaluate the permeation of ibuprofen from thermally activated in situ gel preparations produced from eutectic mixtures intended for rectal administration [64]. Fortunately, the literature reports similarities between the sheep and human rectal mucosae due to epithelia comprising single columnar cell layers [212]. Ham and co-workers reported the development of a microbicide gel for the prevention of human immunodeficiency virus transmission that is suitable for both vaginal and rectal applications. This microbicide gel was tested by collecting colon tissues from gastrointestinal surgery patients with no involvement of inflammatory conditions to demonstrate the efficacy of the gel in restricting viral entry into the rectal mucosa due to proven rectal permeation by the microbicidal agent [65]. This also highlights the importance of utilizing human tissue harvested during surgical procedures that provide a reliable representation of a healthy physiological environment, as harvested tissue may be corrupted with disease that can influence permeation enhancement.

## 7. Transdermal Route of Drug Administration

The skin provides a promising site of administration for systemic drug delivery due to its large surface area and high accessibility [213]. Topical and transdermal drug delivery can be referred to as skin-mediated drug delivery. The main difference is the drug target site, as topical delivery intends to impede drug delivery into the systemic circulation to provide a localized therapeutic effect within the skin layers. Meanwhile, transdermal drug delivery aims to cross the dermal barrier to reach the systemic circulation. Skin-mediated drug delivery has been described as an attractive alternative to the oral route of drug administration due to its numerous advantages [214]. These advantages include achieving systemic drug delivery while bypassing first-pass metabolism and maintaining controlled and long-term drug release, as well as improved patient compliance due to the non-invasive nature of this route [215,216]. In addition, topical drug delivery can benefit localized therapeutic action in targeted skin layers while circumventing side effects associated with systemic therapy [217].

As the rate-limiting step, skin-mediated drug delivery is hindered by the outer skin layer, namely the stratum corneum (SC), which acts as a gatekeeper to prevent the entry of external substances through the skin [218]. Structurally, the SC consist of dead keratinocytes and lipophilic ceramides which form a brick-and-mortar arrangement rendering a dense, lipophilic barrier to drug permeation. Briefly, skin-mediated drug delivery is facilitated by two main pathways, namely the transappendageal and transepidermal pathways [214,219]. The transappendageal pathway (i.e., shunt pathway) refers to drug permeation via hair follicles, sweat glands, and sebaceous glands, which allows the entry of ions and some hydrophilic molecules [219], while the transepidermal pathway is mainly utilized to facilitate dermal drug permeation [220]. Dermal drug permeation can occur via intercellular or transcellular permeation. Intercellular permeation is preferred by hydrophilic drugs and small molecules that can diffuse through the intercellular spaces of the SC lipids to reach the underlying epidermis [214]. On the other hand, the transcellular route allows the crossing of hydrophobic drugs via diffusion into the lipid building blocks of the SC [214].

Effective crossing of the protective SC barrier is hallmarked as a major challenge in the field of topical and transdermal drug delivery [213,215,216,218,220]. Fortunately, several studies have reported active and passive strategies to overcome this formidable barrier [219]. Active strategies involve the utilization of external energy sources to drive dermal permeation by disrupting the SC lipid arrangement or by creating microchannels to cross the SC [220,221]. In contrast, passive dermal permeation may be achieved with the use of either chemical or natural permeation enhancers such as fatty acids, ionic liquids, and surfactants [222,223,224]. Active methods provide a rapid onset of drug permeation, but their use is restricted due to cost and complexity [214,218]. Interestingly, it has been stated that more than 350 molecules have demonstrated effective skin penetration enhancement via different mechanisms [225].

Skin-mediated drug delivery has evolved from first-generation drug delivery systems such as gels, sprays, creams, and ointments, where dermal permeation predominantly relies on passive diffusion. The extent of dermal permeation when using first-generation drug delivery systems is generally governed by specific drug attributes such as low molecular weight (<500 Da) and log P values ranging between 1 and 3 to allow skin-mediated drug delivery [226]. Hence, for a drug to be considered suitable for transdermal drug delivery, it should demonstrate adequate solubility in water and lipids to permit breaching of the lipophilic SC, followed by permeation into the underlying hydrophilic layers [227]. On the other hand, second- and third-generation delivery systems can enhance skin drug penetration by modification of the SC barrier through reversible SC lipid disruption while avoiding damage to the underlying skin layers. Second-generation SC-crossing techniques include drug–vehicle interaction, liposomes and analogues, vesicles, chemical/natural permeation enhancers, energy-driven skin-mediated drug delivery, and bypassing of the SC with devices like microneedles [214,228]. Skin-mimicking models are crucial for replicating the different skin layers to provide foundational insights into permeation mechanisms facilitating successful transdermal and topical drug delivery [56].

### 7.1. In Vito Cell Culture Models for the Assessment of Skin Drug Permeation Enhancement

The most common cell culture systems utilized in dermatological and cosmetic research are two-dimensional monocultures of keratinocytes alone or in combination with fibroblasts [71]. These cell cultures offer advantages such as simplicity, high availability, relatively low costs, and high throughput with rapid result turnover [56]. However, these models do not accurately mimic the complexity of the dermal structure, where fibroblasts and keratinocytes coexist in a hydrophilic environment referred to as the hydrophobic cornified envelope [229]. To improve the skin-mimicking capacity of cell-based in vitro studies, three-dimensional models have been developed and commercialized. These models are composed of either an epidermis or an epidermis layered over a gelatin-based matrix harboring fibroblasts [230]. Even though two-dimensional models are routinely employed during the testing of dermal dosage forms, three-dimensional models have emerged with many improvements and benefits over conventional models. The HaCaT cell model is an example of an immortalized human keratinocyte cell model that is frequently used in two-dimensional models mimicking in vivo dermal characteristics [56,72]. Its rapid proliferation, reliable reproducibility, and simplified adaptability are positive aspects motivating the continued use of these models [56,231,232]. Cell-based in vitro models have been used successfully to find the balance between effective skin drug penetration enhancement and dermal toxicity. However, some two-dimensional cell culture models are suboptimal due to the absence of an SC-mimicking layer. A recent study described the development of a model of improved physiological comparability by adding a hydrophobic barrier to cover the surface of routinely used two-dimensional skin cell culture monolayers [71]. The results revealed that the addition of an SC substitute barrier reduced the cytotoxicity of the generally employed cosmetic solvent, dimethyl isosorbide, and altered the bioactivity of added active components such as oligomeric proanthocyanins and magnesium ascorbyl phosphate, demonstrating that their effectiveness depends on their ability to cross the lipophilic SC (refer to Table 1) [71].

Scaffold-free and scaffold-based three-dimensional-engineered in vitro skin models have been developed to provide in vitro models that represent the in vivo environment more closely [73]. Scaffold-free three-dimensional models, such as cell spheroids, are known for their reduced costs and offer advantages such as reproducibility; however, they lack structural resemblance of tissues such as the position of skin cells needed for a full-thickness stratified epidermis [233]. Conversely, scaffold-based three-dimensional skin models can closely resemble human skin tissue in terms of structure, mechanical presentation, and function. These three-dimensional bioengineered constructs can utilize either natural or synthetic polymers or a combination of both types of polymers [234]. Reconstructed human skin models have been widely used for barrier function and drug permeability studies [74,75,235]. These models are highly reproducible, easily accessible for high-throughput studies, and show improved consistency between study results. However, the main drawback of these models is the lack of a vascular system by which nutrients and oxygen can be provided to the tissue, which is required for prolonged tissue survival. However, a recent publication thoroughly discussed the advancements of vascularizing scaffold-based three-dimensional in vitro skin models to improve their skin-mimicking potential [73]. One of the main challenges of conventional three-dimensional skin in vitro models is the bioengineering of the three-dimensional multilayer skin architecture that aims to imitate all general human skin functions [56,236]. Recently, three-dimensional bio-printing of skin constructs and skin-on-a-chip models have emerged as potential three-dimensional skin model candidates [56,236,237,238].

Overall, the literature concludes that reconstructed human epidermis (RHE) models like EpiSkin^TM^, SkinEthic^TM^, and EpiDerm^TM^ (produced from NHEK cell lines) do not exactly mimic dermal permeation [239,240,241]. As an example (refer to Table 1), a study was performed to investigate the toxic effects of environmental chromium exposure [242]. EpiDerm^TM^ was unable to mediate the crossing of chromium required to mimic the breaching of the SC [242]. However, the literature reports systemic toxic effects following dermal chromium exposure, implying that chromium can pass through the SC [243,244]. Moreover, a recent study comparing the dermal permeation of resorcinol concluded that EpiSkin^TM^ rendered drastically different permeation profiles compared to excised human skin and that the artificial skin-mimicking Strat-M^®^ would be a better skin-mimicking choice than EpiSkin^TM^ [240].

It is important to consider that RHE models are developed to evaluate either dermal permeation or skin irritancy [56]. As an example, the permeation and skin irritancy models of EpiSkin^TM^ are distinguished by their phospholipid composition as the penetration enhancement evaluation model has a significantly reduced phospholipid content compared with the irritancy model [56]. This is understandable as extensive lipid disruption is linked to skin irritancy [245,246,247]. Therefore, it is imperative to consider that skin permeation studies can often provide misleading results if the skin irritancy potential is not evaluated, as excessive dermal lipid disruption can facilitate increased permeation enhancement and dermal irritancy [56,248].

### 7.2. Ex Vivo Models for the Assessment of Skin Drug Permeation Enhancement

Dermatomed human skin remains the gold standard for assessing skin-mediated drug delivery [56,76,77,78,249]. Research revealed that carefully managed frozen human skin offered insights into the passive penetration of substances when skin viability and metabolic activity were not taken into consideration [250]. Interestingly, dermatomed human skin produced greater variability within and between samples compared with excised porcine ear skin [56,251]. This variability was attributed to differences among the donors involved in skin harvesting, such as their age, gender, and ethnicity. Moreover, the type of plastic surgery procedures performed, such as amputations compared with abdominoplastic surgery, also impacted the quality of the skin samples. Additionally, skin harvested from different body parts or regions of cadavers, such as the abdomen, chest, and back, also provided variability in drug permeation [56,252,253,254].

Reports from organizations reported a significant correlation between animal and human ex vivo skin drug permeation [56]. Researchers have used ex vivo skin models to assess drug delivery through and into the skin, as well as for studying the physicochemical determinants of percutaneous penetration. Pig skin is frequently utilized to substitute human skin due to its physiological similarities, involving hair follicle density, Langerhans cells, rete ridges, and the thickness of the SC and its composition, which includes elements like glycosphingolipids and ceramides. Moreover, the thickness of the viable epidermis and the organization of the collagen fibers in the porcine dermis bear a close resemblance to human skin [56,66]. Other animal species that have been used in dermatological research include primates, guinea pigs, rabbits, bovines (specifically utilizing udder tissue), and reptiles (utilizing shed snake skin) [56]. However, the type of animal species has been shown to impact drug permeability due to physiological differences [56,66,255].

A recent study demonstrated the importance of the vehicle used to facilitate skin-mediated drug delivery by establishing different flux values for colchicine presented in a hydrogel, nanoemulsion, emulgel, and nano-emulgel despite its ideal physicochemical properties (refer to Table 2) [227]. Co-solvents are frequently used during the formulation of skin-mediated drug delivery systems to increase drug solubilization, in addition to providing a skin permeation enhancement. For instance, a recent study showed that co-solvents such as ethanol and diethylene glycol monoethyl ether could enhance the permeation of cannabis extract formulated in gels across the skin [256]. Both of these co-solvents easily permeated into and through the SC, resulting in a reversible alteration of the dermal solubility parameters, rendering the skin partitioning of phytocannabinoids [256]. Furthermore, the rapid passage across the SC of these co-solvents resulted in the well-known “pull effect” or “solvent drag” to achieve dermal permeation [256]. On the other hand, combining first-generation skin-mediated drug delivery systems with active skin permeation enhancement methods like microneedles can improve the dermal permeation of drugs previously considered unsuitable for skin-mediated drug delivery [257]. This was confirmed by a recent study utilizing microneedles to improve the dermal permeation of lidocaine incorporated into an ointment via pretreatment with microneedles to create channels across the SC, allowing lidocaine to reach the upper part of the dermis [257]. Interestingly, the same study reported that lidocaine formulated in a nanostructure lipid carrier gel generated a similar skin penetration enhancement without microneedle application [257]. Therefore, nano-based skin-mediated drug delivery approaches have proven effective in skin drug permeation enhancement due to the capacity of these carriers to easily cross the SC and accumulate in adipose tissue supporting the dermis, thereby generating a depo effect [258].

Levamlodipine was successfully incorporated into a transdermal patch to facilitate hypertension therapy while reducing dermal irritation that negatively impacts patient adherence via ion pair formation (Table 2) [259]. Apart from ion pair formation, the inclusion of ionic liquids can also improve dermal permeation by targeting specific lipid components within the skin [260]. As an example, Paraskevopoulos and co-workers suggested that the hydrophobic mismatching achieved by the C24 acyl chain, 1-methyl-3-octylimidazolium bromide (C8MIM), and 3-dodecyl-1-methylimidazolium bromide (C12MIM) rendered affinity for the sterol-rich region of the skin barrier, thereby establishing targeted lipid disruption [260]. Hydrophobic mismatching is attributed to an imbalance in hydrocarbon chain lengths presented by the different lipid building blocks of the skin, thereby implying that hydrophobic mismatching can successfully cause dermal lipid disruption by targeting specific lipid components based on their hydrocarbon chain length [260,261]. Additionally, it was observed in the same study that isotropic phase formation indicated that lipid fluidization of the sterol fraction facilitated the solubilization of other skin lipids potentially impeding the skin barrier [260]. However, ionic liquids can lead to ion pair formation with drugs, as was demonstrated with diclofenac, which rendered unaffected dermal diclofenac permeation when delivered with the help of ionic liquids [260].

Traditional second-generation skin-mediated drug delivery systems like liposomes are currently subjected to modern innovations to generate dual functionalities such as electrostatic interactions and enhanced flexibility of the liposomal lipid bilayer to enhance dermal permeation [262,263]. Variations of liposomes can be created, as demonstrated in a recent study where menthosomes comprising menthol were created, which aided in the delivery of ibuprofen [262]. Carriers can also be produced with surfactants, giving rise to carriers like spanlastics, known for their elasticity and the inclusion of edge activators to facilitate dermal permeation enhancement [264,265].

After performing ex vivo studies, the literature reports employing confocal laser scanning microscopy to clarify results rendered by Franz cell diffusion studies [56]. Confocal laser scanning microscopy allows visualization of skin permeation enhancement by evaluating fluorescein distribution into skin samples to facilitate tracking of skin permeation mechanisms, penetration depth, and whether homogenous dermal drug distribution was achieved [266]. Confocal laser scanning microscopy provides advantages such as non-invasive evaluation and reasonable time consumption [266]. A recent study confirmed the enhanced transcellular permeation of mirtazapine from long-acting transdermal patches via confocal laser scanning microscopy [267]. Fluorescein markers revealed that in the absence of penetration enhancers and counterions, fluorescein could permeate approximately 10 µm. After adding fatty acids to achieve ion pair formation, fluorescein could be detected at a penetration depth of approximately 15 µm. Furthermore, by adding Plurol 15^®^ Oleique CC 497 to formulations comprising fatty acids, a deeper skin penetration enhancement was observed, reaching a depth of up to 30 µm depending on the carbon chain length of the incorporated fatty acid [267]. Interestingly, confocal laser scanning microscopy is also useful when studying dermal permeation from the appendageal pathway as cell-based in vitro skin-mimicking models lack the inclusion of hair follicles. Confocal images highlighting increased fluorescence at the hair follicles can signify reservoir formation within the follicles to gain access to the dense blood capillary network surrounding hair follicles while bypassing the SC, thereby rendering enhanced dermal drug permeation. A recent study reported this reservoir-forming phenomenon for chitosan nanoparticles loaded with curcumin to demonstrate increased dermal permeation with confocal laser scanning microscopy [268].

**Table 1:** Summary of in vitro cell culture-based models used to evaluate drug permeation enhancement across physiological barriers of different routes of drug administration.

Route	Tissue/Cell Line	Drug/Compound	Delivery System/Dosage Form	Mechanism of Permeation Enhancement	Outcomes	Reference
Intranasal	RPMI2650 * cell line	FD-4 *	Co-administration with bio-enhancer (*Aloe vera*, *Aloe ferox*, and *Aloe muth-muth* materials)	Tight junction modulation	Increased P_app_ *	[22]
Nose to brain	RPMI2650 * cell line	TGF-β *	PLGA * or lecithin nanoparticles-in-microparticles containing TGF-β * with mannitol	Opening of tight junctions	Similar transport percentage for TGF-β *	[269]
Nose to brain	Co-culture with RPMI2650 * and human vascular endothelial cells	Insulin	Uncoated and chitosan- coated insulin-loaded solid lipid nanoparticles and PLGA * nanoparticles	Modulated tight junction integrity	Increased P_app_ * from nanoparticle carrier system	[21]
Nose to brain	hCMEC/D3 *	Insulin	Uncoated and chitosan- coated insulin-loaded solid lipid nanoparticles and PLGA * nanoparticles	Modulated tight junction integrity	Insulin solution incorporated into nanoparticles showed a higher P_app_ *	[21]
Nose to brain	Co-culture with RPMI2650 * and human vascular endothelial cells	Meloxicam	In situ thermo-gelling nanoparticle formulation using poloxamer 407	Decreased mucociliary clearance	Enhanced permeation	[95]
Nose to brain	Caco-2 *cell line	Clozapine	Colloidal systems (niosomes and chitosomes)	Tight junction modulation, protection against enzymatic degradation	Formulation systems increased P_app_ * vs. solution	[97]
Buccal	Dialysis membrane	Tedizolid phosphate	Chitosan nanoparticle- loaded buccal film	Bio-adhesion	Controlled release	[270]
Buccal	TR146 * cell line	Carvedilol	Self-assembled liposome from core–sheath nanofibers	Self-assembled liposome from spun fibers after aqueous contact	Improved permeation	[126]
Buccal	TR146 * cell line	[^14^C]-mannitol, FITC-dextran *, FITC-LKP *, and [^3^H]-octreotide	Permeation enhancers such as bile salts, sodium deoxycholate, and glycodeoxycholate	Increase in paracellular flux	Increase in P_app_ *	[271]
Buccal	FNB6-TERT * cell line	Clobetasol-17 propionate	Electrospun polymeric mucoadhesive patches	Mucoadhesion	Sustained release	[123]
Sublingual	Human primary gingival keratinocyte two-dimensional cell culture and spheroid models	Insulin or semaglutide	Novel peptides (lipid-conjugated protamine and a protamine dimer)	Increased pore formation on the cell surface	Increased permeation	[4]
Pulmonary	Calu 3 * cells on air–liquid interface	flu-Na *	Fatty acid bio-enhancers	Paracellular transport modulator	Decrease in TEER * and increase in paracellular transport	[138]
Pulmonary	Calu 3 * cell line cultured on air–liquid interface	Beclomethasone dipropionate salbutamol sulphate	Nanoparticles Amino acid coating	Increased contact area and cytocompatibility	Increased permeation	[37]
Pulmonary	Calu 3 * cell line cultured on air–liquid interface	FD-4 *	Liposome	Tight junction modulation, paracellular transport	Significant decrease in TEER * and P_app_ *	[36]
Pulmonary	Calu 3 * cell line cultured on air–liquid interface	Prednisolone fludrocortisone acetate	Cyclodextrin complex and nanosized amino acid crystal coating	Solubility enhancer	Significant increase in initial drug permeation; thereafter higher, but no significant permeation	[272]
Pulmonary	16HBE14o * cell line cultured on air–liquid interface	Theophylline budesonide	PLA * nanoparticles, co-encapsulation of hydrophilic and lipophilic drugs	Increased contact area, reduced size promoted paracellular transport	No significant changes in transport	[38]
Pulmonary	VA10 * cell line cultured on air–liquid interface	FD-4 *	Chitosan and quaternized derivatives, mucoadhesion and bio-enhancer	Increased contact time, improved paracellular transport	Significantly decreased TEER * and increased P_app_ *	[273]
Pulmonary	Calu 3 * cell line cultured on air–liquid interface	Naringenin flavonoid	HP-β-CD * inclusion complex	Solubility enhancer	Lower cumulative transport, lower efflux	[40]
Pulmonary	Cellulose membrane in three-dimensional-printed diffusion cells	Meloxicam	Nanoparticles	Solubility enhancer, increased contact area, increased cellular uptake	Significant increase in diffusion and flux	[39]
Oral (GIT * and duodenum)	Caco-2 * cell line, HT29-MTX * cell line, Raji B * cells	Insulin	Insulin nanoparticles, insulin in solution	Triple culture	Significantly higher permeation for triple-culture model	[49]
Oral (GIT * and duodenum)	Caco-2 * cell line, HT29-MTX * cell line, Raji B * cells	Insulin	Polystyrene nanoparticles	Triple culture	Size-dependent nanoparticle permeation across both models	[49,193]
Oral (intestinal mucosa)	Three-dimensional bio-printed villi	Metoprolol, atenolol, colchicine	Colchicine permeability by transcellular pathway, P-gp	Three-dimensional intestinal villi compared to flattened villi model	Increased permeability for bio-printed villi model	[196]
Oral (intestinal mucosa)	Gut-on-a-chip system	FITC-dextran *	FD20 * model compound, TEER *, paracellular transport	Transwell^®^ vs. microfluidic model	Higher permeability through paracellular transport in the microfluidic device	[199]
Rectal	EpiVaginal™ *	IQP-0528 *	DuoGel™	Comparing rectal permeation of different DuoGel^TM^ formulations	Consistent permeation rate of IQP-0528 *, highest permeation by IQB3002 *	[65]
Rectal	Caco-2 * cell line	5-fluorouracil	Thermo-reversible gelling film	Intracellular transport	Increased drug solubility and intracellular transport	[63]
Rectal	Caco-2 * cell line	Dapivirine	Nanoparticle vs. free form	Model comparison	Increased permeation	[210]
Topical	Normal human dermal fibroblasts and HaCaT * keratinocyte cell line	Ascorbic acid derivative, dimethyl isosorbide, magnesium ascorbyl phosphate, oligomeric proanthocyanidins/ levan biopolymer	Skin-relevant solvents	Excipient and SC * lipid interaction, drug and SC * lipid interaction	Unsuccessful permeation due to SC * substitute layer	[71]
Topical	EpiSkin^TM^ (NHEK * cell line)	Resorcinol	Aqueous solution, hydrogel, oil-in-water emulsion	Hydration to facilitate SC * lipid disruption	Significant differences between the solution and emulsion in EpiSkin^TM^ and excised human skin permeation	[240]
Topical	EpiDerm^TM^ (NHEK * cell line)	Potassium dichromate and chromium chloride hexahydrate	Chromium aqueous solutions	Mimicking toxin exposure	Chromium is unable to cross the artificial SC *	[242]

* 16HBE14o: human bronchial epithelial cell line; Caco-2: human intestinal carcinoma cell line; Calu 3: human lung adenocarcinoma cell line; EpiVaginal™: normal, human-derived vaginal-ectocervical epithelial and dendritic cells; FD20: fluorescein isothiocyanate-dextran; FD-4: FITC-dextran 4400; FITC: fluorescein isothiocyanate; FITC-LKP: fluorescently labeled peptide; flu-Na: fluorescent marker; FNB6-TERT: immortalized buccal keratinocytes; GIT: gastrointestinal; HaCaT: immortalized human keratinocyte cell line; hCMEC/D3: cell line model of the human blood–brain barrier; HT29-MTX: sub-strain of the colorectal carcinoma-derived cell line HT29; HSA: human serum albumin; HP-β-CD: hydroxypropyl-β-cyclodextrin; IQB3002: formulation comprising IQP-0528; IQP-0528: non-nucleoside reverse transcriptase inhibitor; NHEK: normal human epidermal keratinocyte; P_app_: apparent permeability; P-gp: P-glycoprotein; PLA: poly-lactic acid; PLGA: poly(lactic-co-glycolic acid); Raji B: human B lymphoblastoid cell line; RPMI 2650: nasal septum carcinoma cells; SC: stratum corneum; TGF-β: transforming growth factor β; TEER: transepithelial electrical resistance; TR146: buccal carcinoma cell line; VA10: bronchial epithelial cell line.

**Table 2:** Summary of ex vivo models to evaluate drug permeation enhancement across different physiological barriers of different routes of drug administration.

Route	Tissue/Cell Type	Drug	Delivery Strategy	Mechanism	Outcomes	Reference
Intranasal	Bovine nasal mucosa	Dimenhydrinate	SEDDS *	Increased drug lipophilicity, increased drug permeation	Statistically significant increase in permeation	[58]
Intranasal	Bovine nasal mucosa	Zaleplon	Nanosizing by preparing nanocrystals	Particle size reduction, improved solubility	Increased permeation	[103]
Intranasal	Sheep (frozen nasal mucosa)	Eletriptan hydrobromide	Mucoadhesive polymers	Increased residence time in the nasal cavity, avoiding nasal mucociliary clearance	Superior sustained-release capabilities	[101]
Intranasal	Frontal parts of the nasal concha of sheep	FD-4 *	Microparticle formulation with thermosensitive chitosan gel, and co-administration with piperine	Increased residence time, modulated tight junctions, bio-enhancer, increased paracellular transport	Increased P_app_ *	[24]
Intranasal	Sheep	FD-4 *	Co-administration with bio-enhancer (*Aloe vera*, *Aloe ferox*, and *Aloe muth-muth*)	Tight junction modulation	Increased P_app_ *	[22]
Nose to brain	Goat nasal mucosa	Bromocriptine mesylate glutathione	Nanoemulsion	Small droplet size that leads to more drug solubilization	Increase in % drug transport, flux, and P_app_ *	[59]
Nose to brain	Goat nasal mucosa	Lamotrigine	Nanoliposome	Transport through the lipophilic olfactory and trigeminal nerve systems	Increased % drug transport from nanoliposome	[83]
Nose to brain	Goat nasal mucosa (adhered fat and mucus-free)	Temozolomide	Temozolomide nano lipid chitosan hydrogel formulations	Tight junction modulation, increased transcellular transport	Increased flux with the nano lipid formulation	[23]
Nose to brain	Goat nasal mucosa	Rotigotine	Mucoadhesive nanoemulsion	Larger particle surface area, protection from chemical and biological degradation, mucoadhesion, tight junction modulation	Increased permeability of the mucoadhesive formulation	[100]
Nose to brain	Porcine nasal mucosa	Coumarin 6-labeled preparations were used instead of huperzine A-carrying preparations	Toothbrush-like microneedle patch, delivering lactoferrin-coated nanocarriers (cyclodextrin-based metal–organic frameworks)	Overcomes nasal ciliary clearance and nasal mucosal barriers, improves solubility, physical stability, and biocompatibility, improving brain targeting	Transnasal flux was significantly higher in both nanocarrier formulations	[274]
Nose to brain	Sheep nasal mucosa	Aripiprazole	Nanoemulsion	Increased drug lipophilicity, small globule size, tight junction modulation, mucoadhesion	Increased P_app_ * and flux	[102]
Nose to brain	Sheep nasal mucosa	Lurasidone	Lurasidone hydrochloride-loaded mixed micelles	Decreased particle size, improved wettability, reduced diffusion layer thickness	Mixed micelle formulation increased flux	[275]
Nose to brain	Sheep nasal mucosa	Melatonin	Loaded in lipid nanocapsules	Formulation of lipid nanocapsules with lecithin (a bio-enhancer)	Greater % cumulative release from lipid nanocapsules	[276]
Nose to brain	Sheep nasal mucosa (including septum)	Octreotide acetate	In situ gel (mucoadhesive)	Mucoadhesion, prolonged retention time	Significantly improved P_app_ *	[277]
Buccal	Porcine buccal mucosa	Carvedilol	Self-assembled liposomes from core–sheath nanofibers	Self-assembled liposomes from spun fibers after aqueous contact	Improved permeation	[126]
Buccal	Porcine buccal mucosa	[14C]-mannitol, FITC-dextran *, FITC-LKP *, and [3H]-octreotide	Bile salts, sodium deoxycholate, and glycodeoxycholate	Increase in paracellular flux	Increase in P_app_ *	[271]
Buccal	Porcine buccal mucosa	Epigallocatechin 3-gallate	Encapsulated nanoparticle-loaded mucoadhesive hydrogel nanocomposite	Mucoadhesion and particle size reduction	Faster permeation	[278]
Buccal	Porcine buccal tissue	Delta-aminolevulinic acid	Natural gum-based emulgels	Mucoadhesion	Increased absorption rate	[279]
Buccal	Porcine buccal mucosa	Cannabidiol	Nanoemulsion	Enhanced drug exposure area and stability	Rapid onset of action	[280]
Buccal	Porcine esophageal mucosa	Cannabidiol	Neat and binary lipophilic and hydrophilic vehicles, permeation enhancers	Thermodynamic activity alteration	Increased permeation	[281]
Buccal	Porcine esophageal mucosa	Cannabidiol	Liquisolid systems	Enhanced drug exposure area, wettability, and solubility	Increased permeation	[282]
Buccal	Goat buccal mucosa	Verapamil hydrochloride	Mucoadhesive bilayer patches with menthol and sodium glycocholate	Mucoadhesion	Increased permeation	[60]
Buccal	Rat buccal mucosa	Rizatriptan benzoate and propranolol hydrochloride	Permeation enhancement with *Aloe vera* gel powder	Co-delivery within a mucoadhesive film	Increased permeation	[68]
Buccal	Chicken pouch membrane	Acyclovir	Lipid nanocapsules in gel form	Mucoadhesion	Increased permeation	[130]
Buccal	Rabbit buccal mucosa	Palonosetron	Mucoadhesive film	Biphasic drug release	Increased flux	[62]
Buccal	Whole porcine cheeks	Clobetasol-17 propionate	Electrospun polymeric mucoadhesive patches	Mucoadhesion	Sustained release	[123]
Sublingual	Porcine sublingual mucosa	Rh-BSA * protein vaccine	Mucoadhesive wafers	Mucoadhesion	Increased permeation	[283]
Sublingual	Sheep sublingual mucosa	Febuxostat	Fast-dissolving film containing a self-nano-emulsifying system	Rapid onset of action	Rapid onset of action, improved permeation	[131]
Pulmonary	Isolated perfused rat lung	Insulin	Hexamer insulin formulation using zinc ions	Improved stability, prevented dissociation of insulin	Delayed lung absorption, less metabolic breakdown of insulin	[163]
Pulmonary	Isolated perfused rat lung	Horseradish peroxidase	Poly-L-arginine coating	Improved absorption, tight junction modulation	Increased absorption rate of horseradish peroxidase, a rapid dose response to poly-L-arginine	[44]
Pulmonary	Isolated perfused rat lung	AZD5423 * budesonide fluticasone furoate fluticasone propionate	Formulation strategies (suspension vs. dry powder),	Changes in physicochemical properties	Suspension formulation rendered significantly increased percentage drug absorbed	[164]
Pulmonary	Isolated perfused rabbit lung	5(6)-carboxyfluorescein	Nanoparticle formulation	Increased contact surface area, increased cellular uptake	Perfusate concentrations over time and the sustained plateau were greater for the solution	[45]
Oral (jejenum and ileum)	GI-TRIS *	Oxytocin FITC *	High-throughput experimental screening	Peptides such as MRP-2 *	Higher predictability of drug absorption by GI-TRIS *	[201]
Oral (intestinal mucosa)	Everted gut sac technique	3 model marker compounds	Antipyrine, mannitol, digoxin	Perfusion of gut sac model vs. in vitro	Higher mean permeability coefficient with ex vivo experiments	[202]
Oral (intestinal mucosa)	Everted gut sac technique	Glucose	Glucose with bromelain as bio-enhancer	Ex vivo permeation of glucose uptake	Increased uptake of glucose	[203]
Oral (intestinal mucosa)	In vitro diffusion chamber system	Berberine	DSPE-PEG * co-polymer	Permeability enhancement action on berberine absorption	Increased permeation	[204]
Rectal	Porcine rectal tissue	Dapivirine	Nanoparticle vs. free form	Model comparison	Increased flux	[210]
Rectal	Porcine rectal tissue	Diltiazem hydrochloride	Rectal gels, drug-loaded microsponges in rectal gels	Mucoadhesion	Increased permeation	[63]
Rectal	Sheep intestinal mucosa	Ibuprofen	Thermally activated in situ gels prepared from eutectic mixtures with menthol in a poloxamer gel	Mucoadhesion, increased drug solubility	Increased permeation	[64]
Rectal	Human colorectal tissue	IQP-0528 *	DuoGel™	Comparing rectal permeation of different DuoGel^TM^ formulations	Potent activity against HIV-1 * due to permeation enhancement	[65]
Topical	Dermatomed human skin	Colchicine	Hydrogel, emulgel, nanoemulsion, and nano-emulgel	Hydration of SCE *, reduced droplet size, film formation resulting in reservoir effect	Flux: nanoemulsion > hydrogel > nano-emulgel > emulgel Nanoemulsion reached systemic circulation	[227]
Transdermal	Full-thickness porcine ear skin	Cannabidiol and delta-9-tetrahydrocannabinol	Hydrogel with co-solvent/terpene (propylene glycol, DEGEE *, ethanol/eucalyptol, menthol)	“pull effect”/ “solvent drag effect”	DEGEE * alone/combination with ethanol: enhanced skin deposition	[256]
Topical	Heat-separated human epidermis	Lidocaine	Nanostructure lipid carrier gel, ointment, three-dimensional-printed solid microneedles	Decreased carrier size, creating channels to cross the SC *	Nanostructure lipid carrier gel had similar permeation to microneedles combined with ointment	[257]
Transdermal	Full-thickness porcine ear skin	Levamlodipine	Ion pair formation (patch)	Altered physicochemical properties of drug	Reduced drug retention in epidermis, reduced skin irritation	[259]
Transdermal	Half-thickness human skin	Theophylline, diclofenac	Applying ionic liquids prior to vehicle application	Fluidizing sterol fraction, solubilization of other skin lipids	Enhanced permeation of theophylline, diclofenac permeation unaffected	[260]
Topical	Abdominal rat skin	Ketoconazole	Stearylamine-elastic liposomes	Improved electrostatic interaction with negatively charged corneocytes	Increased permeation	[263]
Transdermal	Wistar Albino rat skin	Ibuprofen	Drug-loaded menthosomes	Menthol: interferes with ceramide hydrogen bonds, formation of microcavities	Significantly increased permeation	[262]
Transdermal	Rat skin	Ferulic acid	Spanlastic vesicles	Decreased particle size, enhanced surface area, skin hydration, receptor-mediated hyaluronic acid transport, SC * hydrophobic interaction	Increased permeation	[284]
Transdermal	Porcine ears	Mirtazapine	Long-acting transdermal patch	Fatty acids, ion pair formation	Increased permeation	[267]
Transdermal	Abdominal mice skin	Curcumin	Chitosan nanoparticles	Zeta-potential change, decreased particle size	Reservoir formation in hair follicles	[268]

* AZD5423: developmental nonsteroidal glucocorticoid; Caco-2: human intestinal carcinoma cell line; DEGEE: diethylene glycol monoethyl ether; DSPE-PEG: 1,2-distearoly-sn-glycero-3-phosphoethanolamine-poly (ethylene glycol); FD-4: FITC-dextran 4400; FITC: fluorescein isothiocyanate; FITC-LKP: fluorescently labeled peptide; GI-TRIS: gastrointestinal tissue robotic interface system; HIV-1: human immunodeficiency virus 1; IQP-0528: non-nucleoside reverse transcriptase inhibitor; MRP-2: multidrug resistance-associated protein 2; NNRTI: non-nucleoside reverse transcriptase inhibitor; P_app_: apparent permeability; P-gp: P-glycoprotein; Rh-BSA: rhodamine B-labeled bovine serum albumin; SC: stratum corneum; SCE: stratum corneum epidermis; SEDDS: self-emulsifying drug delivery system.

## 8. Discussion

An in-depth understanding of physiological barriers to drug delivery is imperative while developing new drug entities, evaluating individual drug absorption mechanisms, and studying permeation enhancement strategies to improve drug bioavailability [285]. The scientific contribution of this review is to consider established and advanced cell culture-based in vitro models and ex vivo models as applied to accurately evaluate permeation enhancement strategies for different routes of drug administration, as the development of in vitro/ex vivo models and permeation enhancement strategies are both as individual topics extensively reviewed elsewhere [10,20,54,56,57,89,261,285,286,287,288,289]. Importantly, different physiological barriers present distinct challenges to drug permeation as these barriers protect the body by governing the crossing of compounds across physiological surfaces such as mucosal membranes and the SC [224,290]. The use of in vitro and ex vivo models is also imperative to determine whether permeation enhancement strategies employed rendered irreversible cell or tissue damage. However, despite the advancements of in vitro and ex vivo model development, the physiologically mimicking models of each extravascular route of drug administration considered by the authors have shortcomings that limit insights into permeation behavior and permeation enhancement strategies due to inadequate mimicking of the in vivo situation.

In terms of evaluating permeation enhancement for the systemic delivery of drugs when intranasally administered, a need exists for the standardization of nasal region-specific models to allow comparison due to morphological differences during the use of tissues from divergent animal species in ex vivo permeation studies. In terms of in vitro cell culture-based studies, the tumorigenic origin and non-organotypic characteristics of these models imply that they cannot completely replace in vivo studies. Moreover, existing and new models for intranasal drug delivery should be fully validated by employing reference agents accompanied by transepithelial electrical resistance measurement and evaluating morphological as well as histological differences [10].

With regard to oromucosal permeation evaluation, a standardized model must be developed to comprehensively consider variables that can impact oromucosal drug delivery such as salivary flux. A histologically and physiologically relevant in vitro–in silico model has been developed to predict chemical permeation across the buccal mucosa [291]. In vitro cell-based models have notoriously produced excellent reproducibility, but have drastically deviated from the in vivo situation. Ex vivo oromucosal models have provided accurate insights into the permeation behavior of drugs. Majid and co-workers have developed and validated an automated ex vivo oromucosal permeation model with a novel Kerski diffusion cell combined with automated sampling [292].

The literature raises the question of whether extrapolating data obtained from Caco-2 cells is sufficient compared with organotypic systems when evaluating permeation enhancement in the pulmonary route of drug administration. The debate continues by stating that technically different pulmonary models should be used to predict drug permeation when considering drug absorption in upper and lower lung regions. Another shortcoming of pulmonary models is the lack of disease-mimicking models to accurately observe the impact that lifestyle choices such as smoking can have on pulmonary drug absorption. Concerns also exist in the literature that bronchial and alveolar epithelial models can exhibit different drug absorption behavior. Therefore, the ideal would be to study large numbers of reference compounds tested in different laboratories to distinguish between the permeation prediction value of Caco-2, alveolar, and bronchial cells [146].

As the most frequently used route of drug administration, the oral route has the most advanced in vitro and ex vivo models mimicking the in vivo situation. However, gastrointestinal disease-mimicking models are lacking to evaluate the extent of permeation influenced by pathology [293]. As an example, numerous models have been developed to imitate irritable bowel disease, but each model presents both advantages and disadvantages [293]. Here, microfluidic gut-on-a-chip systems and organoids can potentially fill the gaps in disease-mimicking models [294]. However, the ideal would be to combine existing models to improve reliability when studying permeation behavior in terms of cost and allowing drugs to reach the market faster. As the last section of the gastrointestinal route of administration, the rectal route has vast potential despite its suboptimal investigation. The development of reliable and practical in vitro and ex vivo models can encourage the advancement of rectal drug delivery systems, which can be very useful during pediatric dosage form development [295].

Finally, skin-mimicking models lack reliable representation of enzymatic activity as excised skin is frozen until needed for diffusion experiments [253]. Therefore, it is important to acknowledge that in silico models have reported strategies to investigate the effect of dermal enzymes on drug metabolism during permeation as similar enzymes can be found in the skin and liver [296]. Disease-mimicking models are also essential for the future as different skin conditions can cause inflammation or impede SC barrier function. This implies that most existing models will not provide adequate information regarding the dermal drug permeation of disease-burdened skin [56]. Another alternative is to combine in vitro cell cultures and ex vivo animal tissue models, as reported by Cappellozza and co-workers [297]. This work reported modifying a bioreactor traditionally hosting cell cultures to instead preserve excised skin by creating a fluid dynamic environment to bridge the gap between in vitro/ex vivo experiments and the in vivo situation. Their morphological and metabolomic findings confirmed that a fluid dynamic environment can preserve excised skin, thereby signifying that ex vivo models can be refined while animal tissue collection can be reduced by re-employing the same tissue sample [297].

Researchers have also attempted to bridge the gap between in vitro/ex vivo models and the in vivo environment by developing or employing existing pharmacokinetic models to describe drug permeation. For instance, drug permeation across the intestines can be impacted by the physiological properties of the membrane itself (i.e., diameter, length, and surface area) and gastric emptying followed by transit in the intestines. The unique physicochemical properties of individual drugs, such as the degree of ionization, lipophilicity, and molecular size, also significantly influence the rate of drug absorption [298]. Therefore, pharmacokinetic models have been proven a useful and cost-effective method for considering drug permeation across different mucosal membranes. Recently, pharmacokinetic models were employed to illustrate the absorption and deposition of methotrexate following nasal administration for targeted delivery to cervical lymph nodes [299], thereby signifying the importance of pharmacokinetic models for simplifying the transition from in vitro, ex vivo, and in vivo studies for future preclinical studies.

Mathematical modeling can also be a useful tool for predicting drug permeation across complex gel networks like mucus. The factors that can influence mucosal permeation are thoroughly described by Cu and Saltzman (2009) [300]. Apart from drug permeation predictions, mathematical models can consider factors such as the deformation of the dosage form, solvent uptake behavior, and diffusion properties. Moreover, kinetic steps can be added to consider the influence of chemically bounded drugs to improve the quality of findings by increasing mathematical model complexity. As an example, a recent publication by Bisotti and co-workers describes mathematical model development to evaluate hydrogels generated from cross-linked polyacrylic compounds to gain insight into the physical properties of hydrogels as a vehicle and their drug delivery capacity [301].

Artificial intelligence predictive models are also receiving increased scientific attention as these models can drastically shorten preclinical periods by evaluating whether a drug is capable of mucosal permeation based on its pharmacochemical properties via machine or deep learning methods. Artificial intelligence models have also been reported to predict the permeation capacity of different dosage forms or excipients [302]. A recent study reported successfully using machine learning techniques to predict dermal drug permeation for microneedle applications by comparing experimental data from other studies to their simulated results. Without performing skin permeation studies, this study was able to investigate the impact of drug loading, microneedle surface area, and time needed to facilitate dermal drug permeation enhancement [303]. However, the value of artificial intelligence must always be weighed against the ethical implications that can arise from utilizing these technologies as human judgement can be bypassed or replaced. This is imperative as ethical considerations govern the appropriate use of in vitro, ex vivo, and in vivo models, and similar considerations must be enforced when employing artificial intelligence models despite the absence of obstacles such as viable tissue collection from human or animal subjects.

## 9. Conclusions

Some newly discovered drug compounds exhibit poor membrane permeation due to their unfavorable physicochemical properties, which consequently results in low bioavailability when administered via extravascular routes of drug administration. Many of these lead compounds, which are highly pharmacologically active, do not progress through all the drug development stages due to insufficient bioavailability. Since efficient delivery of a drug molecule to the site of action is a pre-requisite for therapeutic efficacy, this has prompted scientists to apply different approaches or strategies to enhance the membrane permeation of these compounds to ensure acceptable bioavailability. In vitro cell culture models and ex vivo tissue models have been used to evaluate these strategies of drug permeation enhancement across different mucosal membranes representing different routes of drug administration. The use of these models ensures that research complies with the 3Rs principle (reduction, refinement, and replacement) from a research ethics point of view. In vitro and ex vivo models present numerous advantages such as a relatively low cost with high-throughput potential in comparison with in vivo models. Due to the limitations of conventional two-dimensional cell cultures such as failure to represent the complex physiological in vivo environment, novel three-dimensional cell cultures and co-cultures have emerged as improved models with higher physiological relevance. Furthermore, microfluidics-based systems containing cell cultures have been developed as advanced systems to investigate drug permeation under more realistic and representative conditions. Ex vivo tissue models are not only physiologically relevant, but also provide the opportunity to use waste tissue from animals slaughtered for non-research purposes (e.g., meat production) or tissue from humans after cosmetic surgery. Despite certain limitations of in vitro and ex vivo models, they have already been used successfully to provide proof of concept for drug permeation enhancement and to elucidate the mechanism of permeation enhancement. Additionally, in vitro and ex vivo models have also been used to evaluate the safety and/or toxicological effects of specific drug permeation enhancement strategies. The progress made so far in the advancement of these models has provided the prospect of improved predictive value and even higher in vivo correlation in the future.
